# Incident benzodiazepine and Z-drug use and subsequent risk of alcohol- and drug-related problems: A nationwide matched cohort study with co-twin comparison

**DOI:** 10.1177/02698811251373069

**Published:** 2025-10-01

**Authors:** Xinchen Wang, Zheng Chang, Yasmina Molero, Kayoko Isomura, Lorena Fernández de la Cruz, Paul Lichtenstein, Ralf Kuja-Halkola, Brian M. D’Onofrio, Patrick D. Quinn, Henrik Larsson, Isabell Brikell, Clara Hellner, Jan Hasselström, Nitya Jayaram-Lindström, David Mataix-Cols, Anna Sidorchuk

**Affiliations:** 1 Department of Clinical Neuroscience, Centre for Psychiatry Research, Karolinska Institutet and Stockholm Health Care Services, Region Stockholm, Stockholm, Sweden; 2 Department of Medical Epidemiology and Biostatistics, Karolinska Institutet, Stockholm, Sweden; 3 Department of Psychological and Brain Sciences, Indiana University, Bloomington, USA; 4 Department of Applied Health Science, Indiana University, Bloomington, USA; 5 School of Medical Sciences, Örebro Universitet, Örebro, Sweden; 6 Department of Global Public Health and Primary Care, University of Bergen, Bergen, Norway; 7 Department of Biomedicine, Aarhus University, Aarhus, Denmark; 8 Department of Neurobiology, Care Sciences, and Society, Karolinska Institutet, Stockholm, Sweden; 9 Academic Primary Health Care Center, Region Stockholm, Stockholm, Sweden; 10 Department of Clinical Sciences, Lund University, Lund, Sweden

**Keywords:** benzodiazepines, Z-drugs, alcohol use disorders, drug use disorders

## Abstract

**Background:**

Despite considerable interest in the consequences of benzodiazepine and benzodiazepine-related Z-drug (BZDR) use, little is known about whether and how initiation of BZDR treatment relates to the development of alcohol- and drug-related problems.

**Aims:**

This study aimed to examine the association of incident BZDR dispensing with subsequent alcohol- and drug-related problems.

**Methods:**

This nationwide register-based study included demographically matched and co-twin control cohorts. Among all Swedish residents aged older than 10 years and BZDR-naïve by 2007, 960,430 BZDR-recipients with incident dispensation in 2007–2019 and without any recorded pre-existing substance-related conditions were identified and matched (1:1) to non-recipients from the general population. Twin BZDR-recipients (*n* = 12,048) were linked to 12,579 unexposed co-twins. Outcomes included alcohol and drug use disorders, poisoning, deaths, and related suspected criminal offences. Flexible parametric survival models estimated outcome risks across up to 14 years of follow-up.

**Results:**

In the demographically matched cohort (60% women, median age at BZDR initiation 51 years), incidence rates in BZDR-recipients and non-recipients (per 1000 person-years) were 5.60 versus 2.79 for alcohol-related and 4.15 versus 1.23 for drug-related problems, respectively. In fully adjusted models, relative risks were increased for alcohol- and drug-related problems (adjusted hazard ratio (95% confidence interval): 1.56 (1.53–1.59) and 2.11 (2.05–2.17), respectively). The risks persisted within the co-twin comparison, different follow-ups, and all additional sensitivity analyses.

**Conclusions:**

BZDR initiation was associated with a small but robust increase in absolute and relative risks of developing alcohol- and drug-related problems. The findings contribute to evidence base for making decisions on BZDR treatment initiation.

## Introduction

Benzodiazepines are effective in alleviating the symptoms of anxiety and insomnia and are recognized as a treatment option for managing acute alcohol withdrawal (Dubovsky and Marshall, 2022). Benzodiazepine-related Z-drugs, structurally similar to benzodiazepines, are widely used for treating insomnia (Terzano et al., 2003). Benzodiazepines and Z-drugs (henceforth “BZDR,” if mentioned together) are generally considered safe and effective when used for a short period of time, as recommended by clinical guidelines (Soumerai et al., 2024). However, the risk of dependence and tolerance increases with prolonged use. Although prescription BZDRs are rarely reported as the primary substance of abuse, they are often involved in polysubstance use (Hirschtritt et al., 2018; Jones et al., 2012; Zhang et al., 2019), particularly with opioids and alcohol (Abrahamsson et al., 2017; Cousins et al., 2011; Xu et al., 2021). Furthermore, the concurrent use of BZDR with other substances has been widely acknowledged as a contributor to fatal overdoses (Jones et al., 2014; Kandel et al., 2017). Nevertheless, studies on BZDR and substance-related problems have focused on individuals with pre-existing substance use disorders (Cousins et al., 2011; Jones et al., 2014; Kandel et al., 2017; Xu et al., 2021; McHugh et al., 2020), leaving it unclear whether BZDR use per se might be associated with the subsequent development of substance misuse. Biological studies have suggested shared pharmacological mechanisms for BZDR and some other substances (Engin, 2022). BZDR achieves its effects through allosteric modulation of gamma-aminobutyric acid type A (GABA_A_) receptors, which are known to modulate the activity of neural circuits involved in the behavioral effects of drugs of abuse, such as reward and reinforcement (Crocetti and Guerrini, 2020; Stephens et al., 2017). Similarly to BZDR, alcohol also enhances GABA_A_ receptor activity (Lobo and Harris, 2008). Further, genetic studies showed that the gene encoding the α2 subunit of the GABA_A_ receptor (GABRA2) may modulate the rewarding effects of BZDRs, alcohol, cocaine and opioids, and thus influence drug-seeking behavior (Mallard et al., 2018). In addition to biological mechanisms, behavioral factors were also suggested to play a role as some individuals may transition from medical BZDR use toward misuse of BZDR, other prescription medications or alcohol and illicit drugs use to seek euphoric effects or self-medicate unresolved symptoms (Fatseas et al., 2009; McHugh et al., 2017, 2020).

Understanding how indicated use of BZDR, particularly among new recipients, is associated with subsequent risk of substance use disorders and related problems could be essential for preventing the development of such conditions among patients who might benefit from BZDR treatment. However, epidemiological research on the topic is scarce. Some studies suggest that BZDR use among individuals undergoing opioid treatment could be a risk factor for transitioning to chronic and high-dose opioid use (Lalic et al., 2018, 2020; Sun et al., 2016), whereas those studies focus specifically on changes in prescribed opioid use in the context of pain management. One retrospective cohort study (Sun et al., 2024) reported that incident BZDR prescription was associated with increased risks of alcohol and drug use disorders, while the findings varied between different substance-related outcomes in two other studies (McCabe et al., 2017, 2023). Although informative about potential associations between BZDR and substance-related problems, those studies focused on self-reported lifetime BZDR use (McCabe et al., 2017, 2023) and specific populations (i.e., patients with chronic non-cancer pain (Lalic et al., 2018, 2020), postoperative pain (Sun et al., 2016), or anxiety (Sun et al., 2024)). The interpretation of observed associations could also be limited by potential residual confounding from genetic and environmental factors shared within families, which are important to address given a familial transmission of substance dependence (Bierut et al., 1998) and possible familial co-aggregation between substance use and the disorders for which BZDR is commonly prescribed (e.g., anxiety disorders, depression disorders, and insomnia; Pasman et al., 2020; Wang et al., 2022).

In the present study, we used nationwide Swedish registers and examined the association between BZDR prescription initiation and the subsequent development of broadly defined alcohol- and drug-related problems that resulted in treatment-seeking in specialist services, deaths, or suspected criminal offences. We used a demographically matched cohort and a co-twin control cohort to mitigate various confounding factors, including shared familial confounding.

## Methods

The study was approved by the Swedish Ethical Review Authority (reference number 2020-06540). The requirement for informed consent was waived because the study is register-based, and all individual data were de-identified.

### Study population

**Figure 1.**
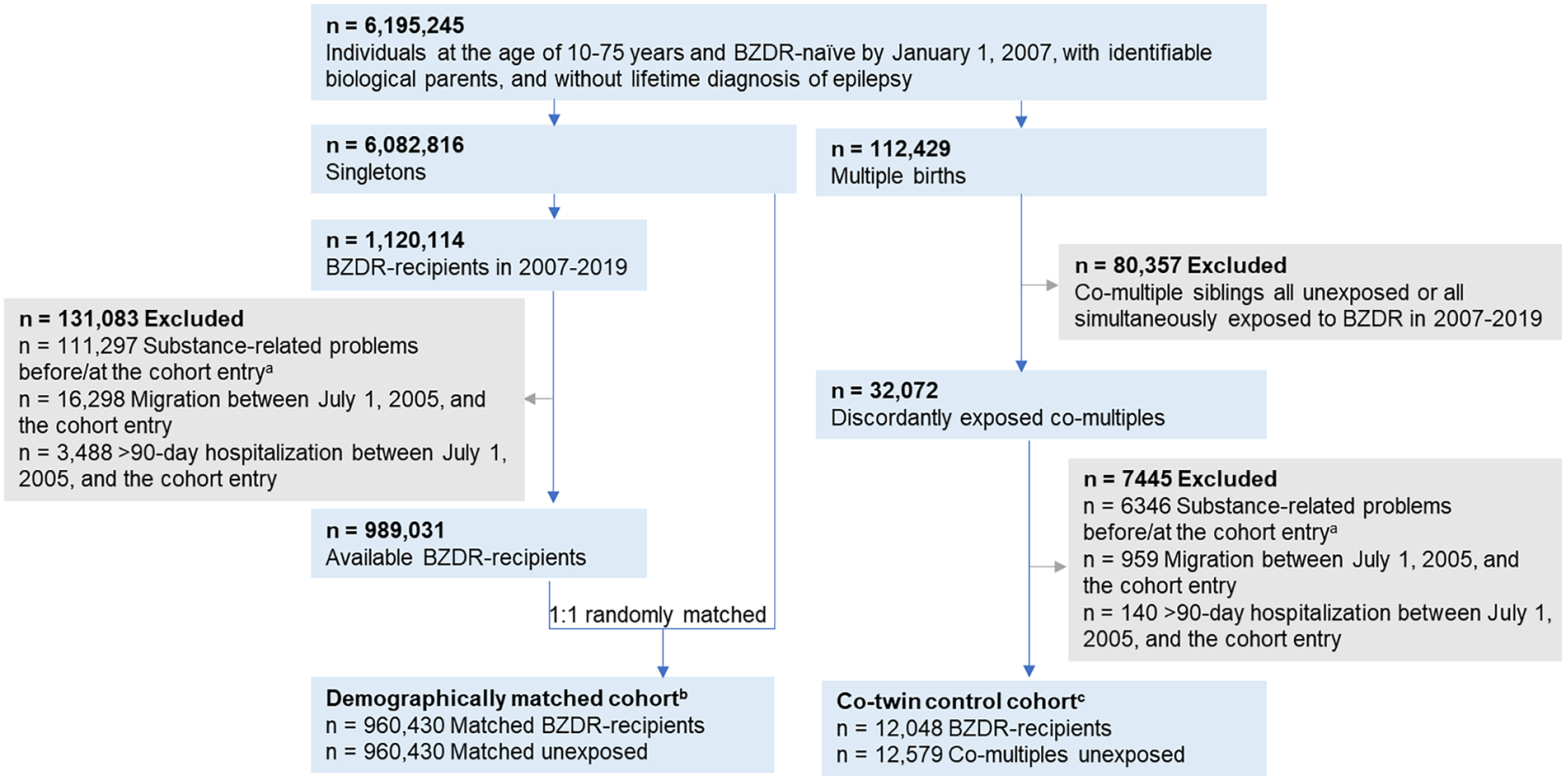
Study design overview: Construction of demographically matched cohort and a co-twin control cohort. ^a^“Cohort entry” refers to the date when the first prescription of any benzodiazepines or related Z-drugs (BZDR) was dispensed to the BZDR-recipient. The same date is assigned as the cohort entry to matched unexposed individuals (in the demographically matched cohort) and to unexposed co-multiples (in the co-twin control cohort). ^b^BZDR-recipients were 1:1 randomly matched by birth year and month, sex, and county of residence at the cohort entry with those who did not have any BZDR dispensation before or at the cohort entry date. To unexposed comparators, the same exclusion criteria were applied as for BZDR-recipients (i.e., no substance-related problems before or at the cohort entry, and no migration or >90-day hospitalization between July 1, 2005, and the cohort entry). ^c^Co-multiples (of which 97.1% were twin pairs) were identified from 12,047 discordantly exposed families.

Using the unique identification numbers assigned to all Swedish residents (Ludvigsson et al., 2009), we linked several nationwide registers with data available through December 31, 2020. The study population included individuals who (1) were 10–75 years old and had no dispensation records of any BZDRs in the Prescribed Drug Register (Wettermark et al., 2007) by January 1, 2007; (2) had identifiable biological parents in the Multi-Generation Register (Ekbom, 2011), and (3) had no lifetime epilepsy diagnosis, according to the National Patient Register (Ludvigsson et al., 2011). The latter was applied since benzodiazepines are commonly used for seizure control (Riss et al., 2008), the prescribing patterns of which may differ from those for individuals with psychiatric conditions. Fulfillment of the abovementioned criteria yielded a study population of 6,195,245 individuals (Figure 1). The Prescribed Drug Register incorporates records of the prescription medications (using Anatomical Therapeutic Chemical (ATC) Classification System codes) dispensed across all pharmacies in Sweden since July 1, 2005. Therefore, all study participants had at least a 1.5-year BZDR dispensation-free washout. The National Patient Register holds inpatient and specialized outpatient care data since 1973 and 2001, respectively, with the diagnoses recorded by the Swedish version of the International Classification of Diseases. The Multi-Generation Register records kinship data for all individuals born in Sweden from 1932 or resided in the country since 1961. Supplemental Note 1 overviews all registers used in the study.

### Study design and exposure to incident BZDR use

We created a demographically matched cohort using the singleton-born individuals from the study population (*n* = 6,082,816; Figure 1). Among them, we selected incident BZDR-recipients as individuals with a first BZDR dispensation recorded in the Prescribed Drug Register in 2007 to 2019 (*n* = 1,120,114) with ATC-codes for benzodiazepine derivatives in anxiolytics (N05BA), hypnotics/sedatives (N05CD), antiepileptics (N03AE), and Z-drugs (N05CF; Supplemental Table S1). The date of the first BZDR dispensation denoted the cohort entry for each BZDR-recipient. We excluded BZDR-recipients if they: (1) were hospitalized for over 90 days between the start of washout period (July 1, 2005) and the cohort entry (this was done to further clarify the exposure status since the Prescribed Drug Register does not cover in-hospital medication); (2) emigrated from or immigrated to Sweden during that period; or (3) had records of any alcohol- or drug-related problems (Supplemental Table S2) before or at the cohort entry (to ensure incident outcome status and mitigate reverse causality). The remaining 989,301 incident BZDR-recipients were eligible for matching to individuals who had no BZDR dispensation record by the cohort entry (i.e., the same date as for BZDR-recipient) and were not excluded due to abovementioned criteria. Matching without replacement was performed 1:1 on birth year and month, sex, and county of residence at the cohort entry, yielding 960,430 pairs of incident BZDR-recipients and their matched unexposed comparators in the demographically matched cohort.

To further control for shared genetic and environmental confounders (D’Onofrio et al., 2013), we constructed a co-twin control cohort using data from the Multi-Generation Register. We identified 112,429 multiple-birth individuals in the study population and assigned a family identifier to each family (Figure 1). Among them, 32,072 co-multiples were discordant by exposure to BZDR, that is, at least one co-multiple in the family had incident BZDR dispensation in 2007–2019 (the date of the first BZDR dispensation was the cohort entry for the exposed co-twin), and the other co-multiple(s) had no BZDR dispensations by the cohort entry (the same date was set as the cohort entry for the unexposed co-twin). After applying the same exclusion criteria as above, we obtained 12,048 BZDR-recipients and their 12,579 unexposed co-multiples (97.1% were twin pairs) from 12,047 families to form the co-twin control cohort.

### Alcohol-related problems and drug-related problems

We defined incident alcohol- and drug-related problems as two distinct outcomes using clinical, pharmacological, criminal, and cause of death data (Quinn et al., 2020). Supplemental Table S2 provides details on outcomes data collection. For “any alcohol-related problems,” we gathered data on alcohol use disorders and unintentional poisoning by alcohol diagnosed in inpatient or specialized outpatient care (main or additional diagnoses) from the National Patient Register, and data on death causes related to alcohol (underlying or secondary causes) from the Cause of Death Register (Brooke et al., 2017). We also retrieved records of dispensed medication for alcohol dependence from the Prescribed Drug Register and alcohol-related suspected criminal offences from the Register of People Suspected of Offences (The Swedish National Council for Crime Prevention, 2019). Similarly, we collected data on “any drug-related problems” including diagnoses of drug use disorders, unintentional poisoning by drugs, death causes related to drug use, dispensed medication for opioid use disorder, and drug-related suspected criminal offences, using the same registers as above (Brooke et al., 2017; Ludvigsson et al., 2011; Wettermark et al., 2007; The Swedish National Council for Crime Prevention, 2019).

### Covariates

For each member of the demographically matched cohort, we collected data on potential confounders in addition to the matching variables (i.e., birth year and month, sex, and county of residence). Disposable family income at the cohort entry (in tertiles) was retrieved from the Longitudinal Integration Database for Health Insurance and Labour Market Studies (Ludvigsson et al., 2019) as a proxy for socioeconomic status. Calendar year of the cohort entry (2007–2009, 2010–2012, 2013–2015, 2016–2019) was included to account for a cohort effect. Furthermore, given the complex interplay between various disorders and substance use, we collected data on the diagnoses from inpatient and specialized outpatient care, recorded in the National Patient Register between 1997 and the cohort entry, for history of psychiatric conditions (other than the outcomes-related) and chronic pain-related conditions (Treede et al., 2015; Supplemental Table S3). Added to that, from the Prescribed Drug Register, we retrieved data on concomitant dispensations (within 3 months before the cohort entry) of other psychotropic, antiepileptic, and analgesic medications (Supplemental Table S1). For the main analyses, three binary variables were constructed for a history of any psychiatric conditions, any pain-related conditions, and having concomitant dispensations. In a sensitivity analysis, each diagnostic group of psychiatric conditions and each type of concomitant medications were used as separate covariates for adjustment. Finally, we collected maternal and paternal lifetime histories of alcohol- and/or drug-related problems, using the same broad definitions as for the outcomes (Supplemental Table S2), and created one binary variable per parent indicating “substance-related problems.” In the co-twin control cohort, the same covariates were used, except birth year and month, and parental lifetime substance-related problems.

### Statistical analyses

In both the demographically matched and co-twin control cohorts, we fitted flexible parametric survival models (Lambert and Royston, 2009) to estimate average and time-varying hazard ratios (HRs) and 95% confidence intervals (CIs) for the association of incident BZDR use with the risk of developing alcohol-related problems and, separately, drug-related problems. Time since the cohort entry was the underlying time scale. In both cohorts, individuals were followed from the cohort entry until the date of the incident outcome, emigration, death by non-outcome, change in exposure status among unexposed (if they were dispensed BZDRs after the cohort entry), or December 31, 2020, whichever came first.

We first ran a minimally adjusted model. In the demographically matched cohort, this model was conditioned on the matching variables, and in the co-twin control cohort, it was conditioned on the family identifier and adjusted for sex. Next, in both cohorts, we applied an adjusted model by additionally controlling for all covariates described above for each cohort. Finally, in a fully adjusted model, we accounted for a possible co-occurrence of alcohol- and drug-related problems by additionally controlling for drug-related problems in the analysis of alcohol-related outcomes, if they occurred during the follow-up, and vice versa. For each outcome, we also calculated crude incidence rate (IR) per 1000 person-years and standardized cumulative incidence (Hinchliffe and Lambert, 2013; controlled for the same covariates as in the fully adjusted model). Moreover, since the associations of interest may change over time, we estimated standardized cumulative incidence, cumulative incidence difference, and fully adjusted time-varying HRs within 1, 3, 5, and 10 years of follow-up. All abovementioned time-varying models had the baseline hazard of 5 degrees of freedom and the effect of BZDR exposure of 3 degrees of freedom.

We conducted three additional analyses using the demographically matched cohort only (due to lack of power in the co-twin control cohort). Supplemental Note 2 describes the methods for each additional analysis. First, we categorized any alcohol-related problems and, separately, any drug-related problems by type of incident outcome event into: (1) non-fatal disorders, (2) unintentional poisoning, (3) deaths, and (4) suspected criminal offences. Specifically for drug-related problems, we also assessed sedatives/hypnotics use disorders and related deaths as an additional type of outcome event of interest. Main analyses were repeated for each type of outcome events using the other types as competing risk. Second, we performed subgroup analyses by participant characteristics and type of initial medication (benzodiazepines or Z-drugs). Third, following a prior study (Rosenqvist et al., 2024), we calculated the cumulative dosage of BZDR by summing up diazepam milligram equivalent doses of each prescription dispensed during the first year after BZDR initiation (Supplemental Table S4), and then assessed the outcome risk by the quartile categories of cumulative BZDR dosage with the follow-up starting from the second year.

We conducted three sensitivity analyses to test the robustness of our findings. Supplemental Note 2 details the methods of sensitivity analysis. First, in the demographically matched cohort, we repeated main analyses for drug-related problems after excluding dispensed medication for opioid use disorders from the outcome definition to avoid possible misclassification for medication used for pain management (Abrahamsson et al., 2017; Quinn et al., 2020). Second, in both the demographically matched and co-twin control cohorts, we re-run a fully adjusted model where each group of psychiatric diagnoses and each type of concomitant medications were included as separate covariates. Third, to further control for confounding by indication (Sendor and Sturmer, 2022), we compared incident BZDR users with incident users of other (apart from BZDR) anxiolytics, hypnotics, and sedatives (ATC-codes: N05B (except N05BA) and N05C (except N05CD and N05CF); Supplemental Table S1). Medication for comparison was chosen due to a partial overlap in indications with BZDR (e.g., anxiety disorders and insomnia). From the study population we identified individuals who collected their first prescription for BZDR or for other (non-BZDR) anxiolytics, hypnotics, and sedatives during 2007–2019, according to the Prescribed Drug Register. Those who initiated both medications simultaneously were excluded. Eligible individuals were categorized as either (1) incident BZDR-recipients or (2) incident recipients of any other anxiolytics, hypnotics, and sedatives, based on the first dispensed medication. For each individual, the cohort entry date was set as the date of the first dispensation of the corresponding medication. Individuals were followed from the cohort entry until the date of outcome, emigration, death by non-outcome, switched exposure status, or December 31, 2020, whichever came first. Crude IR estimations and fully adjusted modeling were performed as described above.

Data management and analyses were performed in SAS version 9.4 (SAS Institute Inc., Cary, NC, USA) and Stata 18.0 (StataCorp LLC, College Station, TX, USA), respectively. All tests employed a two-tailed significance set at *p* < 0.05.

## Results

### Description of the cohorts

In the demographically matched cohort of 960,430 incident BZDR-recipients and 960,430 matched unexposed comparators, 60.0% were women, and the median age at the cohort entry was 51 years (interquartile range (IQR): 37–65). Compared with unexposed individuals, higher proportions of BZDR-recipients had clinical diagnoses from specialist services of any psychiatric conditions (20.3% vs 8.5%) and chronic pain conditions (44.6% vs 34.6%), concomitant medications dispensations (48.1% vs 9.9%), and records of maternal (3.9% vs 2.8%) and paternal (8.9% vs 7.0%) substance-related problems (Supplemental Table S5).

In the co-twin control cohort of 12,048 incident BZDR-recipients and 12,579 unexposed co-twins, 61.1% and 52.0% were women, respectively, and the median age at the cohort entry was 50 years (IQR: 34–63). Compared to their unexposed co-twins, BZDR-recipients more often had psychiatric conditions (20.4% vs 8.8%), chronic pain conditions (42.1% vs 32.9%), and co-medication (49.3% vs 10.6%). Maternal and paternal substance-related problems were present in over 3% and over 7% of all co-twins, respectively (Supplemental Table S5).

### Incident BZDR use and alcohol-related problems

**Figure 2.**
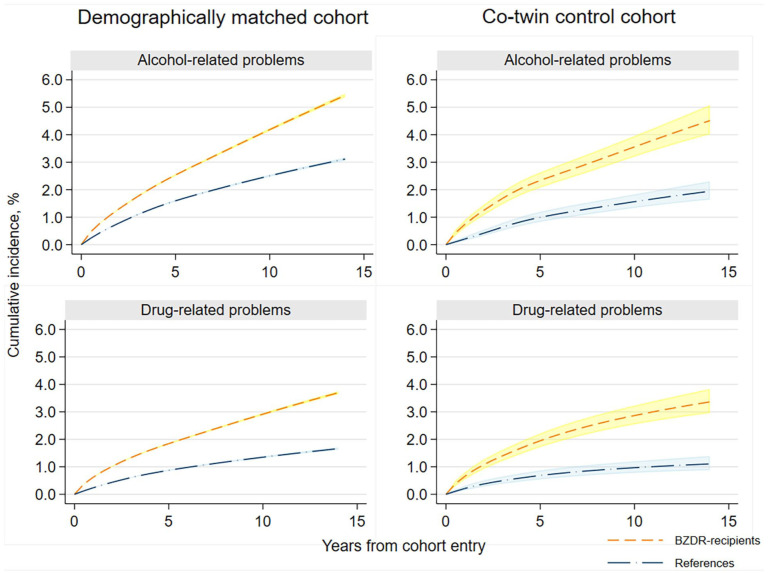
Standardized cumulative incidence and 95% CI of any alcohol-related and drug-related problems as a function of time since the cohort entry among BZDR-recipients and their matched comparators (references) in the demographically matched and co-twin control cohorts. *Note.* Cumulative incidence measures are standardized, that is, controlled for the covariates which were included in the fully adjusted model for the corresponding cohort, and estimated from the flexible parametric model. Shadowed areas denote 95% CI. BZDR: benzodiazepine or related Z-drugs; CI: confidence intervals.

**Figure 3.**
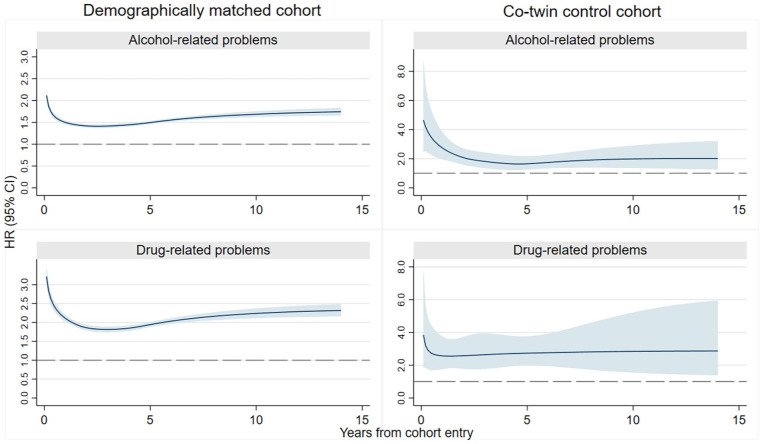
The risk of any alcohol-related and drug-related problems as a function of time since the cohort entry among BZDR-recipients and their matched comparators (references) in the demographically matched and co-twin control cohorts. *Note.* All reported HRs represent the results of fully adjusted model for the corresponding cohort. Shadowed areas denote 95% CI. BZDR: benzodiazepine or related Z-drugs; CI: confidence intervals; HR, hazard ratio.

In the demographically matched cohort, we identified incident events of any alcohol-related problems among 40,475 BZDR-recipients (out of 960,430) and 18,581 matched unexposed individuals (out of 960,430; crude IR 5.60 vs 2.79 per 1000 person-years). Over the whole follow-up, this corresponded to a minimally adjusted HR (aHR) of 2.07 (95% CI: 2.03–2.10), which attenuated in the fully adjusted model (aHR: 1.56; 95% CI: 1.53–1.59; Table 1). In each follow-up period, the standardized cumulative incidence of outcome in BZDR-recipients was small but constantly higher than that in unexposed individuals (e.g., at 1-year follow-up: 0.78% vs 0.44%, risk difference of 0.35% (95% CI: 0.33%–0.37%); at 10-year follow-up: 4.19% vs 2.51%, risk difference of 1.68% (95% CI: 1.62%–1.74%); Table 2 and Figure 2). The relative risk also remained increased in each follow-up period, with aHRs (95% CIs) varying from 1.50 (1.45–1.54) at 1-year follow-up to 1.69 (1.62–1.75) at 10-year follow-up (Table 2 and Figure 3). In the co-twin control cohort, incident events of alcohol-related problems were identified in 438 BZDR-recipients (out of 12,048) and 170 unexposed co-twins (out of 12,579; crude IR 4.75 vs 1.89 per 1000 person-years). The increased relative risk persisted in all models, including the fully adjusted model (aHR: 2.15, 95% CI: 1.76–2.61; Table 1). The time-varying patterns of absolute and relative risks were similar to those of the demographically matched cohort (Table 2, Figures 2 and 3).

**Table 1.** Associations of incident BZDR use with the subsequent risk of any alcohol-related problems and any drug-related problems in 960,430 BZDR-recipients and 960,430 matched comparators in the demographically matched cohort, and among 12,048 BZDR-recipients and 12,579 unexposed co-twins in the co-twin control cohort.

	No. of events, *n* (%)		Crude incidence rate (95% CIs), per 1000 person-years		Minimally adjusted model^a,b^	Adjusted model^c,d^	Fully adjusted model^e^
Study outcomes	BZDR-recipients	Unexposed individuals	BZDR-recipients	Unexposed individuals	HR (95% CI)	HR (95% CI)	HR (95% CI)
Demographically matched cohort							
Any alcohol-related problems	40,475 (4.21)	18,581 (1.93)	5.60 (5.55–5.66)	2.79 (2.75–2.83)	2.07 (2.03–2.10)	1.60 (1.57–1.63)	1.56 (1.53–1.59)
Any drug-related problems	30,228 (3.15)	8227 (0.86)	4.15 (4.10–4.20)	1.23 (1.20–1.25)	3.55 (3.46–3.63)	2.17 (2.11–2.23)	2.11 (2.05–2.17)
Co-twin control cohort							
Any alcohol-related problems	438 (3.64)	170 (1.35)	4.75 (4.31–5.20)	1.89 (1.61–2.18)	2.75 (2,30–3.29)	2.21 (1.81–2.69)	2.15 (1.76–2.61)
Any drug-related problems	360 (2.99)	90 (0.72)	3.88 (3.48–4.28)	1.00 (0.79–1.20)	4.22 (3.35–5.31)	2.87 (2.23–3.71)	2.78 (2.15–3.59)

BZDR: benzodiazepines and related Z-drugs; CI: confidence intervals; HR: hazard ratio.

a In the demographically matched cohort: Conditioned on matching variables (birth year and month, sex, county of residence at the cohort entry).

b In the co-twin control cohort: Conditioned on family identifier and adjusted on sex of each co-twin.

c In the demographically matched cohort: Is additionally adjusted for calendar year at the cohort entry, disposable family income, history of any psychiatric conditions, chronic pain conditions, concomitant dispensations of other psychotropic, antiepileptic, and analgesic medications, if dispensed within 3 months before the cohort entry, and history of maternal and paternal substance-related problems.

d In the co-twin control cohort: Additionally adjusted for the same covariates as the demographically matched cohort, excluding birth year and month, and maternal and paternal substance-related problems.

e In both cohorts: Additionally adjusted for the events of the “other” outcome if it was recorded during the study follow-up (i.e., controlling for drug-related problems in the analysis of alcohol-related problems, if occurred during the follow-up, and vice versa).

**Table 2.** Associations of incident BZDR use with the risk of any alcohol-related problems and any drug-related problems among the members of the demographically matched cohort and the co-twin control cohort at different follow-up periods.

	Years since cohort entry							
	Demographically matched cohort				Co-twin control cohort			
Study outcomes	1 year	3 years	5 years	10 years	1 year	3 years	5 years	10 years
Any alcohol-related problems								
HR (95% CI)^a^	1.50 (1.45–1.54)	1.42 (1.37–1.46)	1.50 (1.46–1.53)	1.69 (1.62–1.75)	2.74 (1.96–3.82)	1.81 (1.36–2.43)	1.65 (1.25–2.18)	1.99 (1.36–2.90)
Cumulative incidence^b^ (95% CI), %								
BZDR-recipients	0.78 (0.77–0.80)	1.76 (1.73–1.78)	2.54 (2.50–2.57)	4.19 (4.14–4.23)	0.74 (0.61–0.89)	1.68 (1.47–1.93)	2.34 (2.07–2.63)	3.56 (3.20–3.95)
Unexposed individuals	0.44 (0.43–0.45)	1.10 (1.07–1.12)	1.60 (1.57–1.63)	2.51 (2.47–2.54)	0.21 (0.14–0.30)	0.64 (0.51–0.79)	1.00 (0.83–1.20)	1.57 (1.34–1.83)
Difference^c^	0.35 (0.33–0.37)	0.66 (0.63–0.70)	0.94 (0.90–0.98)	1.68 (1.62–1.74)	0.53 (0.37–0.69)	1.05 (0.78–1.31)	1.34 (1.00–1.68)	1.99 (1.53–2.45)
Any drug-related problems								
HR (95% CI)^a^	2.11 (2.02–2.19)	1.81 (1.74–1.89)	1.94 (1.88–2.01)	2.24 (2.12–2.37)	2.57 (1.76–3.75)	2.64 (1.77–3.94)	2.74 (1.99–3.76)	2.85 (1.55–5.21)
Cumulative incidence^b^ (95% CI), %								
BZDR-recipients	0.63 (0.61–0.64)	1.34 (1.32–1.36)	1.84 (1.82–1.87)	2.92 (2.88–2.95)	0.65 (0.54–0.80)	1.40 (1.21–1.62)	1.95 (1.72–2.21)	2.87 (2.55–3.23)
Unexposed individuals	0.24 (0.23–0.25)	0.61 (0.59–0.63)	0.87 (0.85–0.90)	1.35 (1.32–1.38)	0.21 (0.15–0.31)	0.50 (0.38–0.65)	0.69 (0.54–0.87)	0.97 (0.78–1.20)
Difference^c^	0.39 (0.37–0.40)	0.73 (0.70–0.76)	0.97 (0.93–1.01)	1.57 (1.51–1.62)	0.44 (0.29–0.60)	0.90 (0.66–1.15)	1.26 (0.96–1.57)	1.90 (1.49–2.31)

*Note*. Cumulative incidence measures in BZDR-recipients and their comparators represent absolute risks among exposed and unexposed members at each specific follow-up time, and cumulative incidence differences represent the corresponding absolute risk differences.

BZDR: benzodiazepines and related Z-drugs; CI: confidence intervals; HR: hazard ratio.

a All reported hazard ratios represent the results of fully adjusted model for the corresponding cohort.

b All reported cumulative incidences are standardized, that is, controlled for the covariates which were included in fully adjusted model for the corresponding cohort.

c Cumulative incidence difference (in percentage) shows the excess outcome cases per 100 individuals in exposed compared to unexposed.

In the first additional analyses, BZDR use was associated with increased risks of all specific types of alcohol-related outcomes, regardless of adjustments (Supplemental Table S6). Within each follow-up period, the standardized cumulative incidence of the specific outcome events was fairly low (e.g., at 10-year follow-up, among the exposed it varied from 0.07% for poisoning by alcohol to 3.36% for alcohol use disorders, and among the unexposed—from 0.04% for poisoning to 1.84% for alcohol use disorders; Supplemental Table S7 and Figure S1). The relative risks for most of the specific events remained increased, with non-significant associations seen only for alcohol-related poisoning at 1-year and death at 1-year, 3-year, and 5-year follow-ups (Supplemental Table S7 and Figure S2). Next, in the subgroup analysis, the associations between BZDR use and any alcohol-related problems persisted in all subgroups, with higher aHRs among women, individuals aged 10–29 years at BZDR initiation, those entering the cohort in 2016–2019, individuals without a history of diagnosed psychiatric conditions, chronic pain conditions or co-medication records, and persons who were initially dispensed benzodiazepines (Supplemental Table S8). In the last additional analysis where cumulative dosage of BZDR use in the first year after BZDR initiation was measured, each category of cumulative dosage was associated with increased relative risk of alcohol-related problems that occurred from the second year after initiation (Supplemental Table S9).

### Incident BZDR use and drug-related problems

In the demographically matched cohort, incident events of drug-related problems were identified in 30,222 BZDR-recipients and 8227 matched unexposed comparators (crude IR 4.15 vs 1.23 per 1000 person-years). For the whole study period, an over 3.5-fold increase in relative risk was observed in the minimally adjusted model (HR: 3.55, 95% CI: 3.46–3.63), which attenuated in the fully adjusted model (aHR: 2.11, 95% CI: 2.05–2.17; Table 1). In each follow-up period, the standardized cumulative incidence of outcome among BZDR-recipients was constantly higher than in unexposed individuals (e.g., at 1-year follow-up: 0.63% vs 0.24%, risk difference of 0.39% (95% CI: 0.37%–0.40%); at 10-year follow-up: 2.92% vs 1.35%, risk difference of 1.57% (95% CI: 1.51%–1.62%); Table 2 and Figure 2)). The relative risk remained elevated in each follow-up period, reaching a fully adjusted aHR of 2.24 (95% CI: 2.12–2.37) at the 10-year follow-up (Table 2 and Figure 3). In the co-twin control cohort, incident drug-related events were seen in 360 BZDR-recipients and 90 unexposed co-twins (crude IR 3.88 vs 1.00 per 1000 person-years), with a fully adjusted aHR of 2.78 (95% CI: 2.15–3.59; Table 1). The time-varying patterns of absolute and relative risks remained similar to those in the demographically matched cohort (Table 2, Figures 2 and 3).

In the first additional analysis of specific types of drug-related outcomes, BZDR use was associated with increased relative risks for all specific outcome events in all models. The most substantial increase in risk was seen for sedatives/hypnotics use disorders and related deaths (aHR: 4.10, 95% CI: 3.71–4.53; Supplemental Table S6). Time-varying risks remained consistently elevated for most specific types of drug-related outcomes (Supplemental Table S7, Figures S3 and S5). Next, in the subgroup analysis, the increased risk of drug-related problems remained in all subgroups, with higher fully adjusted HRs in individuals aged 10–29 years at BZDR initiation, with the cohort entry in 2016–2019, those without a history of diagnosed psychiatric conditions, chronic pain conditions or co-medication records, and individuals who were initially dispensed Z-drugs (Supplemental Tables S10). Lastly, a dose-response association was observed between the cumulative BZDR dosage and the risk of any drug-related problems (Supplemental Table S9).

### Sensitivity analyses

First, focusing on any drug-related problems as an outcome and the demographically matched cohort, the analysis showed that exclusion of dispensation records for opioid use disorders medication from the outcome definition did not alter the results (Supplemental Table S11). Second, in both the demographically matched and co-twin control cohorts, when each group of psychiatric conditions and each type of co-medications were included in the models as separate covariables, the results remained similar to those in the main analyses where psychiatric conditions and co-medications were combined into two binary variables (Supplemental Table S12). Lastly, when comparing incident BZDR-recipients with incident recipients of any other anxiolytics, hypnotics, or sedatives, BZDR use remained associated with increased risks of alcohol- and drug-related problems (Supplemental Table S12).

## Discussion

In this population-based matched cohort and co-twin controlled study of individuals aged 10 years and older and without any recorded pre-existing substance use-related conditions, incident BZDR use was associated with an increased risk of developing severe alcohol- and drug-related problems throughout the whole 14-year study period. After controlling for various potential confounders and shared familial factors, incident BZDR-recipients had 2.11-fold and 2.78-fold higher risks of subsequent alcohol- and drug-related problems, respectively, compared to non-recipients. The increase in relative risks persisted within different follow-up periods and in all subgroup and sensitivity analyses. Also, the risks remained elevated for the specific types of outcome events, that is, disorders, unintentional poisoning, suspected criminal offences, and deaths related to alcohol and drug use. Furthermore, for drug-related problems, a dose-response relationship was observed between cumulative BZDR dosage in the first year after initiation and increased risks of future drug-related outcomes, while for alcohol-related problems, each category of cumulative BZDR dosage was associated with increased outcome risks, but no clear dose-response relationship appeared. Finally, although the estimated IRs of the outcomes were fairly low, these reflect severe conditions that led to hospitalizations or specialist care, suspected criminal offences, and deaths; therefore, even a small increase in absolute and relative outcome risks warrants careful consideration.

To our knowledge, this study is the first to prospectively assess the time-varying associations of incident BZDR use and a broad range of alcohol- and drug-related problems. Although direct comparisons with previous literature are difficult due to the scarcity of studies, the average HRs estimated in our study were comparable to those in a retrospective cohort study where incident benzodiazepine use was associated with a 3-fold increased risk of alcohol and drug use disorders (Sun et al., 2024). The slightly higher risk estimate in that study could probably be explained by the restriction to patients with anxiety and the focus on chronic benzodiazepine use (Sun et al., 2024).

Our findings hold significant clinical importance and could contribute novel insights to existing research. First, by estimating the cumulative incidence difference, we found that for every 50 BZDR-recipients, there was approximately 1 additional case of any alcohol-related problems and 1 additional case of drug-related problems 10 years after initiation. This underscores the need for clinical monitoring of substance use behavior among BZDR-recipients. Second, our results suggested that the associations of incident BZDR use with alcohol- and drug-related problems may not be fully explained by sociodemographic and clinical characteristics, family history of substance-related problems or shared genetic and environmental factors. Third, our assessment of the outcomes extended beyond diagnosed substance use disorders and overdoses, including also alcohol- and drug-related deaths and suspected criminal offences. Since deaths and offences do not depend on treatment-seeking behavior, our study provides a more comprehensive evaluation of substance-related risks. Fourth, we explored the role of BZDR characteristics (type of drugs, dosage) and observed similarly strong associations with outcomes in individuals with benzodiazepines as first medication dispensed and in those with Z-drugs as the initial medication. This aligns with existing evidence suggesting that Z-drugs could also be associated with an increased risk of substance misuse, particularly to drug overdose (Abrahamsson et al., 2017). In the analysis of cumulative BZDR dosage during the first year of BZDR treatment, we observed an increased risk of both alcohol- and drug-related problems already at the lower category of the cumulative dosage, and, moreover, for drug-related problems, a dose-response relation was noted. Fifth, we observed an increased relative risks of both alcohol- and drug-related problems in individuals with a recorded history of psychiatric conditions, chronic pain conditions, or co-medication and in individuals without such records. Although these findings cannot fully rule out confounding by indication, our results suggest that BZDR initiation could be associated with an increased risk of subsequent substance-related problems beyond its association with underlying mental disorders. Moreover, our attempt to further control for confounding by indication via comparing outcome risks in incident BZDR-recipients to incident recipients of any other anxiolytics, hypnotics, or sedatives, showed a persisted increase in risk of alcohol- and drug-related outcomes associated with BZDR.

It is important to mention that while the observational nature of this study precluded inferring causality for the observed associations of BZDR treatment with elevated risks of alcohol- and drug-related outcomes, our findings highlight the need for prescribers to consider monitoring and screening strategies for possible changes in patients’ substance use behaviors throughout the treatment process. Furthermore, one challenge related to the domain of this study is how to understand the context in which BZDR initiation may result in the risk of substance-related problems. While the initiation is an important aspect of the course of BZDR therapy, future studies with appropriate designs to measure the exposure duration, such as a nested case-control design or a cohort study with time-varying assessment of treatment duration, are warranted to further explore whether the duration of treatment impacts the risk. Also, the assessment of the individual BZDR may be taken into account because some benzodiazepines may have stronger toxicity in overdose.

### Strengths and limitations

The study strengths include using population-based Swedish registers with nationwide coverage and standardized data collection that ensured the results generalizability to the national level and minimized the risk of reporting, selection, and information bias. Also, the large sample size, long follow-up time, and a variety of health and administrative data enabled us to analyze alcohol- and drug-related problems separately and perform a range of informative additional and sensitivity analyses. Moreover, we included suspected criminal offences and deaths in the study outcomes to capture substance-related problems that occur in the non-medical context or could go untreated. Furthermore, the use of the co-twin control design allowed to account for confounding due to unmeasured genetic and environmental factors shared within families.

Several limitations should be acknowledged. First, the diagnoses of alcohol and drug use disorders (which constituted most outcome events) may only represent severe cases that led to hospitalization or specialist treatment, leaving milder clinical cases uncaptured. Second, it was uncertain whether and when the BZDR-recipients consumed the dispensed medication. However, prior literature (Benard-Laribiere et al., 2017) suggests that BZDR are mainly used in proximity to their dispensation date, making the register records a reasonable proxy for actual BZDR consumption. Third, data on exposure were solely based on the Prescribed Drug Register. According to surveys from the Swedish Council for Information on Alcohol and Other Drugs, the use of sedatives/hypnotics (mainly BZDRs) without doctor’s prescription is reported by 2% of respondents aged 17–84 years (Sundin et al., 2018) and 4%–5% of late adolescents (Zetterqvist, 2018). Thus, the observed associations could be biased if illegal BZDR purchases or recreational use were disproportionally distributed between the exposure categories. Fourth, we could not fully account for lifestyle factors and their changes over time. The results from the co-twin control cohort might have partly addressed this concern because, by design, it controls for possible confounding by early life environments clustered within families. However, there are still many factors that are specific to each twin/multiple birth individual. Also, when controlling for history of psychiatric and chronic pain-related conditions, we only captured conditions that led to hospitalization or specialist outpatient visit, while the corresponding information from the primary care was unavailable. Although we also attempted to capture underlying diagnoses by collecting the records of concomitant use of other psychotropic, antiepileptic, and analgesic medications, we acknowledged that the proportions of clinical diagnoses were underestimated. Fifth, the potential confounding by contraindication should be noted since clinical guidelines advise against prescribing BZDR to individuals with past or current substance misuse (The Swedish Medical Products Agency, 2025; The Swedish National Board of Health and Welfare, 2019). Our study population was initially restricted to individuals without records of prior substance-related problems; yet, we may have missed pre-existing substance use behaviors not diagnosed/recorded in the registers. Finally, the results may not be generalizable to other countries due to possible differences in BZDR prescribing and the identification of substance-related events.

## Conclusions

The present study suggests an overall small but robust increase in risk of subsequent development of alcohol- and drug-related morbidity, mortality, and suspected criminal offences after BZDR treatment initiation. The associations were not fully explained by sociodemographic characteristics, history of psychiatric conditions, chronic pain conditions, concomitant use of other medications, parental substance-related problems, and shared familial factors. While further research is needed to uncover the mechanisms behind the observed associations, our findings underscore the need for informed decision-making in initiating and monitoring BZDR treatment.

## Supplemental Material

sj-docx-1-jop-10.1177_02698811251373069 – Supplemental material for Incident benzodiazepine and Z-drug use and subsequent risk of alcohol- and drug-related problems: A nationwide matched cohort study with co-twin comparison Supplemental material, sj-docx-1-jop-10.1177_02698811251373069 for Incident benzodiazepine and Z-drug use and subsequent risk of alcohol- and drug-related problems: A nationwide matched cohort study with co-twin comparison by Xinchen Wang, Zheng Chang, Yasmina Molero, Kayoko Isomura, Lorena Fernández de la Cruz, Paul Lichtenstein, Ralf Kuja-Halkola, Brian M. D’Onofrio, Patrick D. Quinn, Henrik Larsson, Isabell Brikell, Clara Hellner, Jan Hasselström, Nitya Jayaram-Lindström, David Mataix-Cols and Anna Sidorchuk in Journal of Psychopharmacology
