# Supplementary material for: Incident benzodiazepine and Z-drug use and subsequent risk of alcohol- and drug-related problems: A nationwide matched cohort study with co-twin comparison
Source: J Psychopharmacol. 2025 Oct 1;40(2):305–15. doi: 10.1177/02698811251373069 (PMC13222384; doi:10.1177/02698811251373069)
Supplement: sj-docx-1-jop-10.1177_02698811251373069 – Supplemental material for Incident benzodiazepine and Z-drug use and subsequent risk of alcohol- and drug-related problems: A nationwide matched cohort study with co-twin comparison [file sj-docx-1-jop-10.1177_02698811251373069.docx]

**Supplementary Information**

**Article title:** Incident benzodiazepine and Z-drug use and subsequent risk of alcohol- and drug-related problems: a nationwide matched cohort study with co-twin comparison

**Journal name:** *Journal of Psychopharmacology*

Xinchen Wang^1^, Zheng Chang^2^, Yasmina Molero^1,2^, Kayoko Isomura^1^, Lorena Fernández de la Cruz^1^, Paul Lichtenstein^2^, Ralf Kuja-Halkola^2^, Brian M D’Onofrio^2,3^, Patrick D Quinn^4^, Henrik Larsson^2,5^, Isabell Brikell^2,6,7^, Clara Hellner^1^, Jan Hasselström^8,9^, Nitya Jayaram-Lindström^1^, David Mataix-Cols^1,10^, Anna Sidorchuk^1^

1. Department of Clinical Neuroscience, Centre for Psychiatry Research, Karolinska Institutet & Stockholm Health Care Services, Region Stockholm, Stockholm, Sweden
2. Department of Medical Epidemiology and Biostatistics, Karolinska Institutet, Stockholm, Sweden
3. Department of Psychological and Brain Sciences, Indiana University, Bloomington, USA
4. Department of Applied Health Science, Indiana University, Bloomington, USA
5. School of Medical Sciences, Örebro Universitet, Örebro, Sweden
6. Department of Global Public Health and Primary Care, University of Bergen, Bergen, Norway
7. Department of Biomedicine, Aarhus University, Aarhus, Denmark
8. Department of Neurobiology, Care Sciences and Society, Karolinska Institutet, Stockholm, Sweden
9. Academic Primary Health Care Centre, Region Stockholm, Stockholm, Sweden
10. Department of Clinical Sciences, Lund University, Lund, Sweden

**Corresponding author:**

Xinchen Wang, Karolinska Institutet, Department of Clinical Neuroscience, Child and Adolescent Psychiatry Research Center, Gävlegatan 22B, floor 8, SE-11330, Stockholm, Sweden, email: [xinchen.wang@ki.se](mailto:xinchen.wang@ki.se)

**Supplementary materials**

**Supplementary Note 1. The Swedish nationwide registers and databases used in the study**

**1)** ***The Swedish Prescribed Drug Register***^1^ encompasses data on prescribed medications dispensed across all pharmacies in Sweden since July 2005 onwards, registered using Anatomical Therapeutic Chemical (ATC) Classification System codes, along with dosage, dispensed amount, dispensation date, and prescriber’s characteristics. The register does not include treatment indication and medications administered in hospitals. In this study, from the Prescribed Drug Register we retrieved data on the initial benzodiazepines and the related Z-drugs (BZDR), and data on other psychotropic, antiepileptic, and analgesic medications, if dispensed within 3 months prior to BZDR initiation. Further, ATC-codes for medication used for alcohol dependence and opioid used disorders were retrieved as a part of the outcome definition, as well as for defining the history of alcohol- and drug-related disorders among the study cohort members, and for parental lifetime substance-related disorders.

**2)** ***The National Patient Register***^2^ captures diagnostic information from somatic and psychiatric inpatient care (covered since 1969 and 1973, respectively) and specialist outpatient care (since 2001), based on the Swedish version of the International Classification of Diseases, Eighth Revision (ICD-8) (1969-1986), ICD-9 (1987-1996), and ICD-10 (1997-onwards). The register was validated for an array of diagnoses with an overall positive predictive value of 85-95% and up to 97% for psychiatric disorders.^3-6^ In this study, data on alcohol- and drug-related disorders and unintentional poisoning were retrieved from this register as a part of the outcome measure. Similar data were used for defining the history of alcohol- and drug-related disorders among the study cohort members, and for parental history of alcohol and drug-related disorders. Further, data on diagnosed psychiatric and somatic conditions, were retrieved from this register to be used as covariates. Finally, the register data were also used to restrict the study population to individuals without the lifetime diagnosis of epilepsy and to gain information on those who were hospitalized for longer than 90 days.

**3)** ***The Cause of Death Register***^7^ includes information on all deaths of Swedish residents, occurring in Sweden or abroad, with dates and the international version of the ICD codes for underlying and contributory causes of deaths since 1952. In this study, death data and causes of death were collected from this register and used a part of the outcome definition (i.e., death due to alcohol and drug use disorders and unintentional poisoning), and death by the cause other than the outcome as a censoring event.

**4)** ***The Total Population Register***^8^ records demographic data of all Swedish inhabitants since 1968, and ***the Migration Register*** - which is part of the Total Population Register - captures migration in and out of Sweden. In this study, data on individual’s age at the first BZDR dispensation, sex, and county of residence in Sweden were collected from the Total Population Register, while information on migration was retrieved from the Migration Register to be used for the inclusion/exclusion criteria and as a censoring event.

**5)** ***The Multi-Generation Register***^9^ contains information on biological and adopted parents of all individuals who were born in Sweden from 1932 onwards or have ever been registered in the country since 1961. With the mother as informant, the father is defined as the mother’s husband at the time of birth, or the man acknowledged as the father by unmarried mothers. The register spans over five generations and contains data on 100% of mothers and 98% of fathers for those born in Sweden since 1961.^9^ In this study, the register was used it to identify and link the biological mothers and fathers to each study participant, and to identify singleton and multiple births.

**6)** ***The Longitudinal Integration Database for Health Insurance and Labour Market Studies*** (LISA, in its Swedish acronym),^10^ since 1990 provides annual socioeconomic data for all Swedish residents aged 16 years and above. In this study, information on a disposable income at the cohort entry year (or the nearest year available) was collected from the LISA register to be used as a covariate.

**7)** ***The Register of People Suspected of Offences***,^11^ since 1995 encompasses records on all individuals aged 15 years or above (the age of criminal responsibility in Sweden) who were suspected of offences after a completed investigation by police, the customs authority, or the prosecution service. The register includes data on all reported offences even if some of them are later found not to have constituted criminal offences. In this study, information on offences related to alcohol or drug use (according to the codes corresponding to the relevant Swedish legislations on alcohol- or drug-related crimes) and the dates of offences were retrieved to be used as a part of the study outcome definition.

The Prescribed Drug Register, the National Patient Register, and the Cause of Death Register are held by the Swedish National Board of Health and Welfare. The Total Population Register, the Multi Generation Register, and the LISA register are held by Statistics Sweden. The Register of People Suspected of Offences is held by the Swedish National Council for Crime Prevention.

**References**

1. Wettermark B, Hammar N, Fored CM, Leimanis A, Otterblad Olausson P, Bergman U, Persson I, Sundstrom A, Westerholm B, Rosen M. The new Swedish Prescribed Drug Register--opportunities for pharmacoepidemiological research and experience from the first six months. *Pharmacoepidemiol Drug Saf* 2007;16(7):726-735.

2. Ludvigsson JF, Andersson E, Ekbom A, Feychting M, Kim JL, Reuterwall C, et al. External review and validation of the Swedish national inpatient register. *BMC Public Health* 2011;11:450.

3. Dalman C, Broms J, Cullberg J, Allebeck P. Young cases of schizophrenia identified in a national inpatient register--are the diagnoses valid? *Soc Psychiatry Psychiatr Epidemiol* 2002;37(11):527-31. doi: 10.1007/s00127-002-0582-3.

4. Ekholm B, Ekholm A, Adolfsson R, Vares M, Osby U, Sedvall GC, et al. Evaluation of diagnostic procedures in Swedish patients with schizophrenia and related psychoses. *Nord J Psychiatry* 2005;59(6):457-464. doi: 10.1080/08039480500360906.

5. Ruck C, Larsson KJ, Lind K, Perez-Vigil A, Isomura K, Sariaslan A, et al. Validity and reliability of chronic tic disorder and obsessive-compulsive disorder diagnoses in the Swedish National Patient Register. *BMJ Open* 2015;5(6):e007520. doi: 10.1136/bmjopen-2014-007520.

6. Sellgren C, Landen M, Lichtenstein P, Hultman CM, Langstrom N. Validity of bipolar disorder hospital discharge diagnoses: file review and multiple register linkage in Sweden. *Acta Psychiatr Scand* 2011;124(6):447-453. doi: 10.1111/j.1600-0447.2011.01747.x.

7. Brooke HL, Talback M, Hornblad J, Johansson LA, Ludvigsson JF, Druid H, et al. The Swedish cause of death register. *Eur J Epidemiol* 2017;32(9):765-773.

8. Ludvigsson JF, Almqvist C, Bonamy AK, Ljung R, Michaelsson K, Neovius M, et al. Registers of the Swedish total population and their use in medical research. Eur J Epidemiol 2016;31(2):125-136.

9. Ekbom A. The Swedish Multi-generation register. *Methods Mol Biol* 2011;675:215-220.

10. Ludvigsson JF, Svedberg P, Olén O, Bruze G, Neovius M. The longitudinal integrated database for health insurance and labour market studies (LISA) and its use in medical research. *Eur J Epidemiol* 2019;34(4): 423-437.

11. The Swedish National Council for Crime Prevention. Available at [www.bra.se](http://www.bra.se)

**Supplementary Note 2. Methods used for conducting additional and sensitivity analyses**

1. **Additional analyses**

Three additional analyses were conducted using the demographically matched cohort only (sample size was insufficient in the twin cohort).

1) First, we categorised any alcohol-related problems and, separately, any drug-related problems by type of incident event into: i) *alcohol use disorders*, including the diagnoses and the records of the dispensed medication for alcohol dependence (or, correspondingly, *drug use disorders*, including the diagnoses and the records of the dispensed medication for opioid use disorder), ii) *unintentional poisoning*, as the diagnoses of poisoning by alcohol (or, correspondingly, by drugs), iii) *deaths* due to alcohol use disorders or unintentional alcohol poisoning (or, correspondingly, due to drug use disorders or unintentional poisoning by drugs), and iv) *suspected* *criminal offences* related to alcohol or drug use. The exact ICD-codes, ATC-codes, and criminal offences codes are reported in **Supplementary Table S2**.

In addition to the abovementioned types of outcome events, we were interested in studying a special type of outcome event that refers to addiction to BZDR. However, due to data availability, we were only able to collect information on sedatives/hypnotics use disorders and related deaths. More precisely, we retrieved information on non-fatal and fatal cases of sedatives/hypnotics use disorders (ICD-10 code: F13.1-13.9) and on non-fatal and fatal cases of acute intoxication/ unintentional poisoning by sedatives/hypnotics (ICD-10 code: F13.0) from the National Patient Register and the Cause of Death Register, respectively. No other data on sedatives/hypnotics-related problems (or specifically on unintentional poisoning by BZDR) were available. For example, for clinical diagnoses with ICD-codes as T42 (poisoning by antiepileptic, sedative-hypnotic and antiparkinsonism drugs) and X41 (accidental poisoning by and exposure to antiepileptic, sedative-hypnotic, antiparkinsonism and psychotropic drugs, not elsewhere classified), further distinction for poisoning by sedative-hypnotic drugs was not possible due to data availability. Therefore, the analysis was based on diagnostic information with ICD-10 code of F13 only. To ensure statistical power for analysis of this type of outcome event, non-fatal and fatal cases of sedatives/hypnotics use disorders, and acute intoxications were combined in one variable.

To analyse an association of incident BZDR use with all abovementioned types of outcome events, we repeated the main analyses for each type of events using the other types of events as competing risk.

2) Second, to further explore the influence of sociodemographic characteristics, history of psychiatric conditions, chronic pain conditions, other medications dispensed in a 3-month proximity to the cohort entry date, and the type of the initial BZDR medication (benzodiazepines [N03AE, N05BA, or N05CD] or Z-drugs [N05CF], which was collected at the first BZDR dispensation in 2007-2019) on associations of interest, we performed subgroup analyses. The analyses were performed for any alcohol-related problems and, separately, for drug-related problems. For each study covariate, differences between subgroup HRs were estimated by introducing interaction terms to the models or using Wald tests.

3) Third, we examined the association between the cumulative dosage of BZDR dispensed during the first year after BZDR initiation and the risk of alcohol- and drug-related outcomes that occurred starting from the second year after treatment initiation. For that, we restricted the initial demographically matched cohort to individuals with the follow-up longer than one year since the cohort entry (i.e., those who during the first year did not develop the outcome of interest or were not censored due to death, emigration from Sweden, end of study or – if unexposed – change of the exposure status). Thus, from the original 960,430 pairs, 59,825 (6.2%) and 62,687 (6.5%) matched pairs were excluded from the analyses of alcohol-related problems and drug-related problems, respectively. Cumulative dosage among BZDR-recipients was determined in two steps (1) by summing up all doses for all BZDRs that individual dispensed during the first year after BZDR initiation, and (2) by converting the cumulative doses into the diazepam milligram equivalent (DME) dose. The conversion was made in line with the prior literature.^1^ **Supplementary Table S4** reports the scale used for DME conversion. The estimated cumulative BZDR dosage was then categorized into quartiles: >0 - <300DME, ≥300 - <900DME, ≥900 - <2300DME, ≥2300DME. Then, we then calculated hazard ratios (HR) and 95% confidence intervals (CI) for any alcohol-related problems and, separately, drug-related problems comparing the BZDR-recipients belonging to cumulative doses categories to their matched comparators (i.e., 0 DME). The follow-up started from the beginning of the second year after the cohort entry until the outcome of interest, death by non-outcome, emigration, change of the exposure status (for unexposed), and the study end, whichever occurred first. With the cumulative dosage from the first study year and the follow-up starting from the beginning of the second year the analysis was an analogy to intention to treat approach. We used full adjustment for modelling.

**2. Sensitivity analyses**

We conducted three sensitivity analyses to test the robustness of our findings.

1) First analysis was performed in the demographically matched cohort (to avid lacking statistical power due to insufficient sample size). We tested the new definition for drug-related problems where we excluded dispensation records for medication used for treatment of opioid used disorders (N07BC01 [buprenorphine], N07BC02 [methadone], N07BC05 [levomethadone], and N07BC51 [buprenorphine, combinations]) from the outcome definition. This was done in line with prior literature to avoid possible misclassification for medication used for pain management.^2,3^ Thus, for individuals from the study population, data on “any drug-related problems” outcome were re-collected using register records of drug use disorders, unintentional poisoning by drugs, death causes related to drug use, and drug-related suspected criminal offences (**Supplementary Table S2**). All models used for the main analyses were re-applied here.

2) Second sensitivity analysis was conducted in both the demographically matched and co-twin control cohorts and referred to the change in the adjustment strategy. Specifically, in fully-adjusted model, we replaced previously used combined binary variable for a history of any inpatient or specialist outpatient diagnoses of psychiatric conditions by 13 binary variables, each for a history of one diagnostic group: (1) Schizophrenia, schizotypal, and delusional disorders, and psychotic disorders (2) Bipolar disorders, (3) Depressive disorders, (4) Anxiety disorders, (5) Obsessive-compulsive disorder, (6) Reaction to severe stress and adjustment disorders, (7) Dissociative, somatoform and other neurotic disorders, (8) Mental retardation, (9) Autism spectrum disorders, (10) Attention Deficit/Hyperactivity Disorder, (11) Disruptive behaviour disorders, (12) Suicide attempt/self-injury, and (13) Nonorganic sleep disorders and organic insomnia (as listed in **Supplementary Table S3**). Likewise, we replaced previously used combined binary variable for any concomitant medication by 8 binary variables, each for one type of co-medication: (1) Antidepressants, (2) Psychostimulants (centrally acting sympathomimetics), (3) Mood stabilisers, (4) Other antiepileptics (excluding benzodiazepine derivatives), (5) Antipsychotics, (6) Other anxiolytics (excluding benzodiazepine derivatives) and other hypnotics and sedatives (excluding benzodiazepine derivatives and Z-drugs), (7) Analgesics (non-opioids), and (8) Opioids (as listed in **Supplementary Table S1**).

3) Third, to further control for confounding by indication,^4^ we compared incident BZDR users with incident users of other (apart from BZDR) anxiolytics, hypnotics, and sedatives (ATC-codes: N05B [except N05BA] and N05C [except N05CD and N05CF]). Important to mention that according to the Swedish National Board of Health and Welfare,^5^ the list of anxiolytics, hypnotics, and sedatives that were eligible for being prescribed in Sweden in 2007-2020 (corresponds to the study period) was restrictive and did not include medication with a high risk of dependence (for example, barbiturates). **Supplementary Table S1** details anxiolytics, hypnotics, and sedatives that were prescribed in Sweden 2007-2020 and, that were selected for this sensitivity analysis. In brief, this includes hydroxyzine (ATC-code: N05BB01), buspirone (N05BE01), melatonin (N05CH01), clomethiazole (N05CM02), propiomazine (N05CM06), valerianae radix (N05CM09). It also includes medications, for which the dispensation records were found in quite a small amount during the study period: meprobamate (N05BC01; less than 400 dispensations per year among the entire Swedish population in 2007-2011 and <20 dispensations/year in 2012-2020), chloral hydrate (N05CC01; <500 dispensations/year in 2007-2020), suvorexant (N05CJ01; no dispensations before 2015 and <70 dispensations/year in 2015-2020), scopolamine (N05CM05; 100-400 dispensations/year in 2007-2013 and <50 dispensations/year in 2014-2020), and hypnotics and sedatives in combination, excluding barbiturates (N05CX; no dispensations before 2011 and <10 dispensations/year in 2011-2020). Medication for comparison was chosen due to a partial overlap in indications between BZDR and abovementioned other anxiolytics, hypnotics, and sedatives (e.g., anxiety disorders and insomnia). From the study population we identified individuals who collected their first prescription for BZDR or for other (non-BZDR) anxiolytics, hypnotics, and sedatives during 2007-2019, according to the Prescribed Drug Register. Those who initiated both medications simultaneously were excluded. Eligible individuals were categorised into being either 1) incident BZDR-recipients or 2) incident recipients of any other anxiolytics, hypnotics, and sedatives, based on the first dispensed medication. For each individual, the cohort entry date was set as the date of the first dispensation of the corresponding medication and denoted the start of follow-up. Individuals were followed until the date of outcome, emigration, death by non-outcome, switched exposure status or December 31, 2020, whichever came first. Crude incidence rate estimations and fully-adjusted modelling were performed the same way as in the main analyses.

**References**

1. Rosenqvist TW, Wium-Andersen MK, Wium-Andersen IK, Jørgensen MB, Osler M. Long-Term Use of Benzodiazepines and Benzodiazepine-Related Drugs: A Register-Based Danish Cohort Study on Determinants and Risk of Dose Escalation. Am J Psychiatry. 2024 Mar 1;181(3):246-254.

2. Abrahamsson T, Berge J, Ojehagen A, Hakansson A. Benzodiazepine, z-drug and pregabalin prescriptions and mortality among patients in opioid maintenance treatment-A nation-wide register-based open cohort study. Drug Alcohol Depend. 2017;174:58-64.

3. Quinn PD, Fine KL, Rickert ME, et al. Association of Opioid Prescription Initiation During Adolescence and Young Adulthood With Subsequent Substance-Related Morbidity. JAMA Pediatr. 2020;174(11):1048-1055.

4. Sendor R, Sturmer T. Core concepts in pharmacoepidemiology: Confounding by indication and the role of active comparators. Pharmacoepidemiol Drug Saf. 2022;31(3):261-269.

5. The Swedish National Board of Health and Welfare. Statistical Database, Pharmaceuticals. Available from: <https://sdb.socialstyrelsen.se/if_lak/val_eng.aspx>

**Supplementary Table S1. ATC-codes retrieved from the Prescribed Drug Register for benzodiazepines, benzodiazepine-related Z-drugs, and other medications**

| **Medication** | **ATC-codes** | **Comments** |
| --- | --- | --- |
| **Benzodiazepines and benzodiazepine-related drugs retrieved from the PDR** | | |
| ***Benzodiazepine derivatives in antiepileptics*** | | |
| Clonazepam | N03AE01 |  |
| ***Benzodiazepine derivatives in anxiolytics*** | | |
| Diazepam | N05BA01 |  |
| Oxazepam | N05BA04 |  |
| Clorazepate | N05BA05 |  |
| Lorazepam | N05BA06 |  |
| Bromazepam | N05BA08 |  |
| Clobazam | N05BA09 |  |
| Alprazolam | N05BA12 |  |
| ***Benzodiazepine derivatives in hypnotics/sedatives*** | | |
| Nitrazepam | N05CD02 |  |
| Flunitrazepam | N05CD03 |  |
| Triazolam | N05CD05 |  |
| Midazolam | N05CD08 |  |
| ***Benzodiazepine-related drugs (Z-drugs)*** | | |
| Zopiclone | N05CF01 |  |
| Zolpidem | N05CF02 |  |
| Zaleplon | N05CF03 |  |
| **Other medications retrieved from the PDR, if recorded during 3 months prior to the cohort entry date** | | |
| Antidepressants | N06A |  |
| Psychostimulants (centrally acting sympathomimetics) | N06BA |  |
| Mood stabilisers | N03AF01, N03AF02, N03AG01, N03AX09, N03AN01 |  |
| Other (non-BZD) antiepileptics (i.e., excluding benzodiazepine derivatives) | N03A (except N03AE)^a^ and (except N03AF01, N03AF02, N03AG01, N03AX09, N03AN01)^b^ | ^a^ Excluded from ‘non-BZD’ antiepileptics and used to select BZD derivatives in antiepileptics  ^b^ Excluded from ‘non-BZD’ antiepileptics and used to select mood stabilizers |
| Antipsychotics | N05A |  |
| Other (non-BZD) anxiolytics (i.e., excluding benzodiazepine derivatives), and other (non-BZDR)-hypnotics/sedatives (i.e., excluding benzodiazepine derivatives and benzodiazepine-related Z-drugs) | N05B (except N05BA)^c^  N05C (except N05CD, N05CF)^d^ | ^c^ Excluded from ‘non-BZD’ anxiolytics and used to select benzodiazepine derivatives in anxiolytics  ^d^ Excluded from ‘non-BZDR’ hypnotics/sedatives and used to select benzodiazepine derivatives in hypnotics/sedatives (N05CD) and benzodiazepine-related drugs (N05CF) |
| ***Among that, medications available to be***  ***used as comparison for sensitivity analysis:*** |  |  |
| Hydroxyzine | N05BB01 |  |
| Meprobamate | N05BC01 | Only few dispensations recorded under the study period |
| Buspirone | N05BE01 |  |
| Chloral hydrate | N05CC01 | Only few dispensations recorded under the study period |
| Melatonin | N05CH01 |  |
| Suvorexant | N05CJ01 | Only few dispensations recorded under the study period |
| Clomethiazole | N05CM02 |  |
| Scopolamine | N05CM05 | Only few dispensations recorded under the study period |
| Propiomazine | N05CM06 |  |
| Valerianae radix | N05CM09 |  |
| Hypnotics and sedatives in combination,  excluding barbiturates | N05CX | Only few dispensations recorded under the study period |
| Analgesics (non-opioids) | N02B, N02C |  |
| Opioids | N02A |  |

*Abbreviations*: ATC, Anatomical Therapeutic Chemical Classification System; BZD, benzodiazepines; BZDR, benzodiazepines and benzodiazepine-related Z-drugs when being mentioned together; PDR, Prescribed Drug Register

**Supplementary Table S2**. **ICD-codes, ATC-codes, and criminal offences codes for the ascertainment of alcohol-related problems and drug-related problems^a^**

| **Alcohol/drug-related problems** | **ICD-10 codes (1997-onwards),**  **ATC-codes (2005-onwards),**  **criminal offences codes (1995-onwards)** | **ICD-9 codes**  **(1987-1996)** | **ICD-8 codes (1969-1986)** |
| --- | --- | --- | --- |
| Alcohol use disorders or deaths | *ICD-10 codes:*  F10.1-F10.9 (Mental and behavioural disorders due to use of alcohol) | *ICD-9 codes:*  291 (Alcoholic psychosis),  303 (Alcoholism/ dependence), | *ICD-8 codes:*  291 (Alcoholic psychosis),  303 (Alcoholism/ dependence) |
| Medications used for alcohol dependence treatment^b^ | *ATC-codes*:  N07BB01 (disulfiram), N07BB03 (acamprosate), N07BB04 (naltrexone), N07BB05 (nalmefene) |  |  |
| Alcohol-related poisoning | *ICD-10 codes:*  F10.0 (Acute intoxication by alcohol)  T51.0 (Toxic effect of ethanol),  X45 (Accidental poisoning by and exposure to alcohol) | *ICD-9 codes:*  305A (Alcohol misuse)  980A (Toxic effect of ethanol) | *ICD-8 codes:*  980,00 (Toxic effect of ethanol),  980,01 (Toxic effect of ethanol surrogates) |
| Alcohol-related criminal offences | *Criminal offences codes*:  3005 (Driving under the influence of only alcohol, or both alcohol and drugs; from the Law 1951:649, §4 and 4a),  3201 (Operating maritime vessel under the influence of alcohol or other drugs; from the Law 1994:1009, chapter 20, §4 and 5) |  |  |
| Drug-related disorders or deaths | *ICD-10 codes:*  F11.1-F11.9 (Mental and behavioural disorders due to use of opioids)  F12.1-F12.9 (...due to use of cannabinoids)  F13.1-F13.9 (...due to use of sedatives or hypnotics)  F14.1-F14.9 (...due to use of cocaine)  F15.1-F15.9 (...due to use of other stimulants)  F16.1-F16.9 (...due to use of hallucinogens)  F18.1-F18.9 (...due to use of volatile solvents)  F19.1-F19.9 (...due to multiple drug use of other psychoactive substances) | *ICD-9 codes:*  292 (Drug-induced psychoses)  304 (Drug dependence) | *ICD-8 codes:*  304 (Drug dependence) |
| Medications used in opioid use disorder treatment^b,c^ | *ATC-codes*:  N07BC01 (buprenorphine), N07BC02 (methadone), N07BC05 (levomethadone), and N07BC51 (buprenorphine, combinations) |  |  |
| Drug-related poisoning | *ICD-10 codes:*  F11.0 (Acute intoxication due to use of opioids)  F12.0 (...due to use of cannabinoids)  F13.0 (...due to use of sedatives or hypnotics)  F14.0 (...due to use of cocaine)  F15.0 (...due to use of other stimulants)  F16.0 (...due to use of hallucinogens)  F18.0 (...due to use of volatile solvents)  F19.0 (...due to use of multiple drug use of other psychoactive substances)  T40 (Poisoning by narcotics and psychodysleptics),  T42 (Poisoning by antiepileptic, sedative-hypnotic and antiparkinsonism drugs)  X41 (Accidental poisoning by and exposure to antiepileptic, sedative-hypnotic, antiparkinsonism and psychotropic drugs, not elsewhere classified)  X42 (Accidental poisoning by and exposure to narcotics and psychodysleptics (hallucinogens), not elsewhere specified) | *ICD-9 codes:*  305X (Narcotic and medication misuse)  969 (Poisoning by psychotropic medications and narcotics)  969G (Poisoning by psychodysleptics) | *ICD-8 codes:*  971 (Poisoning by narcotics) |
| Drug-related criminal offenses | *Criminal offences codes*:  3070 (Driving under the influence of only drugs; from the Law 1951:649, paragraphs 4 and 4a),  5010 (Possession of drug only; from the Narcotic Drug Act 1968:64),  5011 (Use of drug only; from the Narcotic Drugs Act 1968:64),  5012 (Possession and use of drugs; from the Narcotic Drugs Act 1968:64) | | |

*Note*: The ICD-10 codes are included in the outcome definition, while together with ICD-8 and ICD-9, the codes are used for constructing variables ‘history of any alcohol use- or drug-related problems prior to the cohort entry’ (used as an additional exclusion criterion), and ‘maternal and paternal lifetime substance-related problems’ (used as a potential confounder).

^a^ ICD-codes are collected from the National Patient Register and the Cause of Death Register for non-fatal and fatal cases, respectively, ATC-codes are collected from the Prescribed Drug Register, and codes for criminal offences are collected from the Register of People Suspected of Offences.

^b^ Medications are selected according to the Swedish Pharmaceutical guidelines (www.fass.se)

^c^ Methadone or buprenorphine which are used in opioid use disorders therapy - namely N07BC01, N07BC02, N07BC05, and N07BC51 – were included in the outcome definition for the main analyses only. In the sensitivity analysis, these medications were excluded from outcome measures due to a possible misclassification between the use of methadone and buprenorphine for OUD therapy and pain therapy.

*Abbreviations*: ATC, Anatomical Therapeutic Chemical Classification System; BZDR, benzodiazepines and benzodiazepine-related Z-drugs; ICD, International Classification of Diseases.

**Supplementary Table S3. Psychiatric and somatic conditions collected from the National Patient Register, if recorded between 1997^a^ and the cohort entry date^b^**

| **Conditions** | **ICD-10 codes** |
| --- | --- |
| ***Psychiatric conditions, sleep disorders, and suicide attempt*** | |
| Schizophrenia, schizotypal, and delusional disorders, and psychotic disorders | F20, F21, F22, F23, F24, F25 (except F25.0), F28, F29 |
| Bipolar disorders | F25.0, F30, F31, F34.0 |
| Depressive disorders | F32, F33, F34 (except F34.0), F38, F39 |
| Anxiety disorders | F40, F41 |
| Obsessive-compulsive disorder | F42 |
| Reaction to severe stress and adjustment disorders | F43 |
| Dissociative, somatoform and other neurotic disorders | F44, F45, F48 |
| Mental retardation | F70-F79 |
| Autism spectrum disorders | F84.0, F84.1, F84.3, F84.5, F84.8, F84.9 |
| Attention Deficit / Hyperactivity Disorder (including medication for ADHD by ATC codes) | F90 and ATC-codes: N06BA01, N06BA02, N06BA04, N06BA09, N06BA12 |
| Disruptive behaviour disorders | F91 |
| Suicide attempt /self-injury (definite or undetermined intent) | X60–X84, Y10–Y34 |
| Nonorganic sleep disorders and insomnia (organic) | F51.0, G47.0 |
| ***Somatic conditions (chronic pain syndrome related conditions)*** | |
| Back and neck pain | M4, M5 |
| Headache | G43, G44, R51 |
| Arthritis, osteoarthritis, and joint pain | M0, M1, M2, R26 |
| Temporomandibular joint disorders | K07.6 |
| Fibromyalgia | M79.7 |
| Other musculoskeletal/connective tissue pain | M3, M6, M7, M8, M9 |
| Gastro-esophageal reflux disease | K21 |
| Irritable bowel syndrome | K58 |
| Chronic fatigue syndrome | G93.3 |
| Interstitial cystitis | N30.1 |
| Sexual pain | F52.5, F52.6, N94, N48.3 |
| ***Lifetime diagnosis of epilepsy used as the exclusion criterion for study population*** | |
| Epilepsy, status epilepticus | G40, G41 |

^a^ Introduction of the International Classification of Diseases, Tenth Revision (ICD-10) in Sweden

^b^ ‘Cohort entry’ refers to the date when the first prescription of any benzodiazepines or related Z-drugs (BZDR) was dispensed to the BZDR-recipient. The same date is assigned as the cohort entry to matched unexposed individual (in the demographically matched cohort) and to unexposed co-twin (in the co-twin control cohort)

*Abbreviations*: ADHD, Attention Deficit / Hyperactivity Disorder; ATC, Anatomical Therapeutic Chemical Classification System; ICD, International Classification of Diseases

**Supplementary Table S4. Diazepam milligram equivalent doses**

| **Benzodiazepines and benzodiazepine-related drugs (BZDR) retrieved from the PDR** | | |
| --- | --- | --- |
| **Benzodiazepine derivatives in antiepileptics** | **ATC code** | **Diazepam equivalent doses, mg** |
| Clonazepam | N03AE01 | 20 |
| **Benzodiazepine derivatives in anxiolytics** |  |  |
| Diazepam | N05BA01 | 1 |
| Oxazepam | N05BA04 | 0.5 |
| Clorazepate | N05BA05 | 0.75 |
| Lorazepam | N05BA06 | 5 |
| Clobazam | N05BA09 | 0.5 |
| Alprazolam | N05BA12 | 10 |
| **Benzodiazepine derivatives in hypnotics/sedatives** |  |  |
| Nitrazepam | N05CD02 | 1 |
| Flunitrazepam | N05CD03 | 0.5 |
| Triazolam | N05CD05 | 40 |
| Midazolam | N05CD08 | 1.5 |
| **Benzodiazepine-related drugs (Z-drugs)** |  |  |
| Zopiclone | N05CF01 | 1.33 |
| Zolpidem | N05CF02 | 0.5 |
| Zaleplon | N05CF03 | 0.5 |

*Note*: Diazepam milligram equivalent doses scale is applied according to the prior literature.^1^

*Abbreviation*: ATC, Anatomical Therapeutic Chemical Classification System; PDR, the Prescribed Drug Register

**Reference**

1. Rosenqvist TW, Wium-Andersen MK, Wium-Andersen IK, Jørgensen MB, Osler M. Long-Term Use of Benzodiazepines and Benzodiazepine-Related Drugs: A Register-Based Danish Cohort Study on Determinants and Risk of Dose Escalation. Am J Psychiatry. 2024 Mar 1;181(3):246-254.

**Supplementary Table S5. Characteristics of incident BZDR-recipients and unexposed individuals included in the demographically matched cohort and co-twin control cohort. Values are numbers (percentages) unless specified otherwise**

| **Characteristics** | **Demographically matched cohort** | | **Co-twin control cohort** | |
| --- | --- | --- | --- | --- |
|  | **BZDR-recipients**  **(n=960,430)** | **Matched unexposed (n=960,430)** | **BZDR-recipients**  **(n=12,048)** | **Unexposed co-twins (n=12,579)** |
| Women | 576,679 (60.0) | 576,679 (60.0) | 7356 (61.1) | 6535 (52.0) |
| Men | 383,751 (40.0) | 383,751 (40.0) | 4692 (38.9) | 6081 (48.0) |
| **Age at the cohort entry, years,**  **median (IQR)** | 51 (37-65) | 51 (37-65) | 50 (34-63) | 50 (34-63) |
| **Age at the cohort entry, years** |  |  |  |  |
| 10-17 | 4867 (0.5) | 4867 (0.5) | 107 (0.9) | 118 (0.9) |
| 18-29 | 134,098 (14.0) | 134,098 (14.0) | 2075 (17.2) | 2184 (17.4) |
| 30-64 | 570,680 (59.4) | 570,680 (59.4) | 7151 (59.4) | 7446 (59.2) |
| ≥65 | 250,785 (26.1) | 250,785 (26.1) | 2715 (22.5) | 2831 (22.5) |
| **Cohort entry year** |  |  |  |  |
| 2007-2009 | 282,969 (29.5) | 282,969 (29.5) | 3817 (31.7) | 4018 (31.9) |
| 2010-2012 | 235,751 (24.5) | 235,751 (24.5) | 3048 (25.3) | 3172 (25.2) |
| 2013-2015 | 220,133 (22.9) | 220,133 (22.9) | 2615 (21.7) | 2716 (21.6) |
| 2016-2019 | 221,577 (23.1) | 221,577 (23.1) | 2568 (21.3) | 2673 (21.2) |
| **Any psychiatric condition^a^** | 195,249 (20.3) | 81,402 (8.5) | 2460 (20.4) | 1110 (8.8) |
| **Chronic pain condition^b^** | 428,758 (44.6) | 331,915 (34.6) | 5076 (42.1) | 4139 (32.9) |
| **Any co-medication^c^** | 461,726 (48.1) | 94,827 (9.9) | 5935 (49.3) | 1332 (10.6) |
| **Maternal substance-related problems^c^** | 37,676 (3.9) | 26,420 (2.8) | 395 (3.3) | 407 (3.2) |
| **Paternal substance-related problems^c^** | 85,008 (8.9) | 67,619 (7.0) | 940 (7.8) | 982 (7.8) |
| **Household disposable income at the cohort entry^d^** |  |  |  |  |
| Lowest tertile | 290,296 (30.2) | 283,735 (29.5) | 3599 (29.9) | 3651 (29.0) |
| 2^nd^ tertile | 288,997 (30.1) | 285,512 (29.7) | 3639 (30.2) | 3609 (28.7) |
| Highest tertile | 281,615 (29.3) | 293,047 (30.5) | 3397 (28.2) | 3854 (30.6) |
| Unknown | 99,522 (10.4) | 98,136 (10.2) | 1413 (11.7) | 1465 (11.6) |
| **Follow-up duration,** **years, median (IQR)** |  |  |  |  |
| Alcohol-related problems | 7.6 (4.2-10.9) | 6.8 (3.6-10.2) | 7.9 (4.3-11.2) | 7.0 (3.8-10.5) |
| Drug-related problems | 7.7 (4.3-11.0) | 6.8 (3.7-10.2) | 7.9 (4.3-11.2) | 7.1 (3.9-10.6) |

^a^ Includes psychiatric disorders (schizophrenia, schizotypal, delusional, and psychotic disorders, bipolar disorders, depressive disorders, anxiety disorders, obsessive-compulsive disorder, reaction to severe stress and adjustment disorders, dissociative, somatoform and other neurotic disorders, mental retardation, autism spectrum disorders, attention deficit/hyperactivity disorder, disruptive behaviour disorders), suicide attempt/self-injury, nonorganic sleep disorders and organic insomnia, if recorded in the National Patient Register between 1997 (start of ICD-10) and the cohort entry date

^b^ Includes conditions related to chronic pain syndrome (back and neck pain, headache, arthritis, osteoarthritis, and joint pain, temporomandibular joint disorders, fibromyalgia, other musculoskeletal/connective tissue pain, gastro-esophageal reflux disease, irritable bowel syndrome, chronic fatigue syndrome, interstitial cystitis, and sexual pain), if recorded in the National Patient Register between 1997 (start of ICD-10) and the cohort entry date

^c^ Includes dispensation records of antidepressants, centrally acting sympathomimetics, mood stabilisers, non-BZD-antiepileptics, antipsychotics, non-BZD-anxiolytics, non-BZDR-hypnotics/sedatives, non-opioid analgesics, and opioids, if dispensed within 3 months before the cohort entry, according to the Prescribed Drug Register

^d^ Missing data on income were marked as unknown and included in the models as nominal variable

*Abbreviation*: BZD, benzodiazepines; BZDR, benzodiazepines and related Z-drugs (if mentioned together); ICD, International Classification of Diseases; IQR, interquartile range

**Supplementary Table S6**. **Associations of incident BZDR use with the risk of developing alcohol-related and drug-related problems subdivided by type of incident outcome events in 960,430 BZDR-recipients and 960,430 matched unexposed individuals in the demographically matched cohort**

|  | **No. of events, n (%)** | | **Crude incidence rate (95% CIs), per 1000 person-years** | | **Minimally-adjusted model^a^** | **Adjusted model^b^** | **Fully-adjusted model^c^** |
| --- | --- | --- | --- | --- | --- | --- | --- |
|  | **BZDR-recipients** | **Matched**  **unexposed** | **BZDR-recipients** | **Matched**  **unexposed** | **HR (95% CI)** | **HR (95% CI)** | **HR (95% CI)** |
| **Any alcohol-related problems** | | |  |  |  |  |  |
| Alcohol use disorders^d^ | 32,926 (3.43) | 13,265 (1.38) | 4.56 (4.51-4.61) | 1.99 (1.96-2.02) | 2.35 (2.30-2.40) | 1.75 (1.71-1.79) | 1.71 (1.67-1.75) |
| Poisoning | 562 (0.06) | 238 (0.02) | 0.08 (0.07-0.08) | 0.04 (0.03-0.04) | 2.23 (1.91-2.59) | 1.59 (1.33-1.89) | 1.54 (1.29-1.83) |
| Offences | 6623 (0.69) | 4862 (0.51) | 0.92 (0.89-0.94) | 0.73 (0.71-0.75) | 1.31 (1.26-1.36) | 1.18 (1.13-1.23) | 1.15 (1.11-1.20) |
| Deaths | 862 (0.09) | 568 (0.06) | 0.12 (0.11-0.13) | 0.09 (0.08-0.09) | 1.42 (1.27-1.58) | 1.24 (1.10-1.39) | 1.21 (1.08-1.37) |
| **Any drug-related problems** | | |  |  |  |  |  |
| Drug use disorders^e^ | 17,040 (1.77) | 3451 (0.36) | 2.34 (2.30-2.38) | 0.51 (0.50-0.53) | 4.70 (4.53-4.88) | 2.54 (2.44-2.65_ | 2.46 (2.36-2.56) |
| Poisoning | 2369 (0.25) | 376 (0.04) | 0.33 (0.31-0.34) | 0.06 (0.05-0.06) | 6.05 (5.43-6.75) | 3.42 (3.04-3.85) | 3.37 (2.99-3.79) |
| Offences | 10,733 (1.12) | 4465 (0.46) | 1.47 (1.45-1.50) | 0.67 (0.65-0.69) | 2.38 (2.30-2.46) | 1.79 (1.72-1.87) | 1.75 (1.68-1.83) |
| Deaths | 732 (0.08) | 185 (0.02) | 0.10 (0.09-0.11) | 0.03 (0.02-0.03) | 3.70 (3.15-4.35) | 2.27 (1.90-2.72) | 2.17 (1.81-2.60) |
| **Additional outcome within drug-related problems** | | | |  |  |  |  |
| Sedatives/hypnotics use disorders^f^ | 4212 (0.44) | 512 (0.05) | 0.58 (0.56-0.60) | 0.08 (0.07-0.08) | 7.81 (7.13-8.56) | 4.26 (3.85-4.70) | 4.10 (3.71-4.53) |

*Note*: Some individuals who were defined as having ‘any alcohol-related problems’ may have more than one type of the outcome event that occurred at the same day (multiple simultaneous outcomes appeared in 1% of BZDR-recipients and in <2% of references). Same refers to individuals with ‘any drug-related problems’ (multiple simultaneous outcomes appeared in 2% of BZDR-recipients and in <3% of references). As a result, those individuals may appear in more than one analysis of the specific type of outcome.

^a^ Conditioned on matching variables (birth year and month, sex, and country of residence at the cohort entry)

^b^ Additionally adjusted for calendar year at the cohort entry, disposable family income, history of any psychiatric conditions, chronic pain conditions, concomitant dispensations of other psychotropic, antiepileptic, and analgesic medications, if dispensed within 3 months before the cohort entry, and history of maternal and paternal substance-related problems

^c^ Additionally adjusted for the events of the ‘other’ outcome if it was recorded during the study follow-up (i.e., controlling for drug-related problems in the analysis of alcohol-related problems, if occurred during the follow-up, and visa verse)

^d^ Including dispensation of medication for alcohol dependence

^e^ Including dispensation of medication for opioid use disorders

^f^ Additional type of outcome event within drug-related problems created by collecting the corresponding records from the National Patient Register and the Cause of Death Register. Due to data availability and in order to avoid underpowered analysis, this covariate combines the records of non-fatal and fatal cases of mental and behavioural disorders due to use of sedatives or hypnotics (ICD-10 codes: F13.1-F13.9) and non-fatal and fatal cases of poisoning by use of sedatives or hypnotics (ICD-10 code: F13.0)

*Abbreviation*: BZDR, benzodiazepines and benzodiazepine-related Z-drugs; CI, confidence intervals; HR, hazard ratio

**Supplementary Table S7**. **Associations of incident BZDR use with the risk of developing alcohol-related and drug-related problems subdivided by type of incident outcome events among 960,430 BZDR-recipients and 960,430 matched unexposed individuals from the demographically matched cohort at different follow-up periods**

|  | **Years since cohort entry** | | | | | | | |
| --- | --- | --- | --- | --- | --- | --- | --- | --- |
|  | **Alcohol-related problems** | | | | **Drug-related problems** | | | |
|  | **1 year** | **3 years** | **5 years** | **10 years** | **1 year** | **3 years** | **5 years** | **10 years** |
| **Disorders *(alcohol use disorders or, correspondingly, drug use disorders)*^a^** | | | | | | | | |
| HR (95% CI)^b^ | 1.65  (1.59-1.71) | 1.54  (1.48-1.59) | 1.62  (1.57-1.67) | 1.83  (1.75-1.91) | 2.57  (2.42-2.73) | 2.14  (2.01-2.27) | 2.20  (2.09-2.31) | 2.38  (2.19-2.59) |
| Cumulative incidence^c^ (95% CI), % |  |  |  |  |  |  |  |  |
| BZDR-recipients | 0.65  (0.64-0.67) | 1.42  (1.40-1.44) | 2.03  (2.01-2.06) | 3.36  (3.32-3.41) | 0.34  (0.33-0.35) | 0.72  (0.70-0.73) | 0.99  (0.98-1.01) | 1.60  (1.57-1.62) |
| Unexposed individuals | 0.32  (0.31-0.33) | 0.80  (0.78-0.82) | 1.16  (1.14-1.19) | 1.84  (1.80-1.87) | 0.11  (0.10-0.11) | 0.26  (0.25-0.28) | 0.39  (0.37-0.40) | 0.62  (0.60-0.64) |
| Difference^d^ | 0.33  (0.31-0.35) | 0.62  (0.59-0.65) | 0.87  (0.83-0.91) | 1.53  (1.47-1.58) | 0.24  (0.22-0.25) | 0.45  (0.43-0.47) | 0.61  (0.58-0.63) | 0.98  (0.94-1.02) |
| **Poisoning *(by a corresponding substance)*** | | | | | | | | |
| HR (95% CI)^b^ | 1.31  (0.96-1.79) | 1.52 (1.14-2.04) | 1.56  (1.23-1.99) | 1.47  (1.11-1.95) | 3.20  (2.71-3.78) | 2.35  (1.98-2.80) | 2.36  (2.03-2.76) | 2.41  (1.85-3.14) |
| Cumulative incidence^c^ (95% CI), % |  |  |  |  |  |  |  |  |
| BZDR-recipients | 0.01  (0.01-0.01) | 0.02 (0.02-0.02) | 0.03  (0.03-0.04) | 0.07  (0.06-0.08) | 0.08  (0.07-0.08) | 0.13  (0.12-0.14) | 0.16  (0.15-0.17) | 0.20  (0.19-0.21) |
| Unexposed individuals | 0.01  (0.00-0.01) | 0.01 (0.01-0.02) | 0.02  (0.02-0.02) | 0.04  (0.03-0.05) | 0.01  (0.01-0.02) | 0.03  (0.03-0.04) | 0.04  (0.04-0.05) | 0.06  (0.05-0.06) |
| Difference^d^ | 0.01  (0.00-0.01) | 0.01 (0.00-0.01) | 0.01  (0.01-0.02) | 0.03  (0.02-0.04) | 0.07  (0.06-0.07) | 0.10  (0.09-0.11) | 0.12  (0.11-0.13) | 0.14  (0.13-0.15) |
| **Offences *(related to use of a corresponding substance)*** | | | | | | | | |
| HR (95% CI)^b^ | 1.08  (1.01-1.15) | 1.13  (1.06-1.20) | 1.18  (1.11-1.24) | 1.24  (1.14-1.35) | 1.72  (1.62-1.83) | 1.58  (1.48-1.68) | 1.74 (1.65-1.83) | 2.02  (1.87-2.19) |
| Cumulative incidence^c^ (95% CI), % |  |  |  |  |  |  |  |  |
| BZDR-recipients | 0.12  (0.11-0.12) | 0.30  (0.29-0.31) | 0.45  (0.44-0.46) | 0.74  (0.72-0.76) | 0.21  (0.20-0.22) | 0.50  (0.49-0.52) | 0.71 (0.70-0.73) | 1.20  (1.17-1.22) |
| Unexposed individuals | 0.10  (0.10-0.11) | 0.26  (0.25-0.27) | 0.38  (0.37-0.39) | 0.58  (0.57-0.60) | 0.11  (0.11-0.12) | 0.29  (0.28-0.31) | 0.42 (0.40-0.43) | 0.66  (0.64-0.68) |
| Difference^d^ | 0.01  (0.01-0.02) | 0.04  (0.02-0.05) | 0.07  (0.05-0.09) | 0.16  (0.13-0.18) | 0.09  (0.08-0.11) | 0.21  (0.19-0.23) | 0.29 (0.27-0.32) | 0.54  (0.50-0.57) |
| **Deaths *(due to use of a corresponding substance)*** | | | | | | | | |
| HR (95% CI)^b^ | 1.10  (0.88-1.38) | 1.09  (0.92-1.29) | 1.17  (0.97-1.41) | 1.40  (1.16-1.69) | 1.46  (1.08-1.97) | 2.14  (1.65-2.77) | 2.71  (2.01-3.65) | 2.67  (1.95-3.66) |
| Cumulative incidence^c^ (95% CI), % |  |  |  |  |  |  |  |  |
| BZDR-recipients | 0.01  (0.01-0.01) | 0.03  (0.03-0.03) | 0.05  (0.05-0.06) | 0.12  (0.11-0.13) | 0.01  (0.00-0.01) | 0.02  (0.02-0.02) | 0.04  (0.03-0.04) | 0.08  (0.08-0.09) |
| Unexposed individuals | 0.01  (0.01-0.01) | 0.03  (0.02-0.03) | 0.04  (0.04-0.05) | 0.08  (0.07-0.09) | 0.00  (0.00-0.01) | 0.01  (0.01-0.02) | 0.02  (0.02-0.02) | 0.03  (0.03-0.04) |
| Difference^d^ | 0.00  (0.00-0.00) | 0.00  (0.00-0.01) | 0.01  (0.00-0.02) | 0.04  (0.03-0.05) | 0.00  (0.00-0.00) | 0.01  (0.00-0.01) | 0.02  (0.01-0.02) | 0.05  (0.04-0.06) |

*Note*: Cumulative incidence measures in BZDR-recipients and unexposed individuals represent absolute risks of outcomes in these groups at each specific follow-up time, and cumulative incidence differences represent the corresponding absolute risk differences.

^a^ For alcohol-related problems (left part of the table): diagnoses of alcohol use disorders also include dispensation records of medication for alcohol dependence; for drug-related problems (right part of the table): diagnoses of drug use disorders alco include dispensation records of medication for opioid use disorder

^b^ All reported hazard ratios represent the results of fully-adjusted model for the demographically matched cohort

^c^ All reported cumulative incidences are standardised, i.e., controlled for the covariates which were included in fully-adjusted model for the demographically matched cohort

^d^ Cumulative incidences difference (in percentage) shows the excess outcome cases per 100 individuals in exposed compared to unexposed

*Abbreviation*: BZDR, benzodiazepines and benzodiazepine-related Z-drugs; CI, confidence intervals; HR, hazard ratio

**Supplementary Table S8**. **Associations of incident BZDR use with the risk of developing any alcohol-related problems stratified by study characteristics in 960,430 BZDR-recipients and 960,430 matched unexposed individuals in the demographically matched cohort**

|  | **No. of events, n (%)** | | **Crude incidence rate (95% CIs), per 1000 person years** | | **Fully-adjusted model^a^** |
| --- | --- | --- | --- | --- | --- |
|  | **BZDR-recipients** | **Matched**  **unexposed** | **BZDR-recipients** | **Matched**  **unexposed** | **HR (95 % CI)** |
| **Sex** |  |  |  |  |  |
| Women | 17,512 (3.04) | 7291 (1.26) | 3.90 (3.84-3.96) | 1.83 (1.79-1.87) | 1.51 (1.46-1.55) |
| Men | 22,963 (5.98) | 11,290 (2.94) | 8.39 (8.28-8.50) | 4.21 (4.14-4.29) | 1.62 (1.58-1.66) |
| **Age at cohort entry, years** |  |  |  |  |  |
| 10-29 | 9579 (6.89) | 3203 (2.30) | 8.94 (8.76-9.12) | 3.16 (3.05-3.27) | 1.79 (1.71-1.88) |
| 30-64 | 26,731 (4.68) | 12,427 (2.18) | 5.80 (5.74-5.87) | 2.94 (2.89-2.99) | 1.59 (1.55-1.62) |
| ≥65 | 4165 (1.66) | 2951 (1.18) | 2.69 (2.61-2.77) | 2.08 (2.00-2.16) | 1.25 (1.19-1.32) |
| **Cohort entry year** |  |  |  |  |  |
| 2007-2009 | 17,567 (6.21) | 9112 (3.22) | 5.50 (5.42-5.58) | 3.28 (3.22-3.35) | 1.35 (1.31-1.39) |
| 2010-2012 | 11,323 (4.80) | 5010 (2.13) | 5.55 (5.45-5.65) | 2.61 (2.54-2.69) | 1.62 (1.56-1.68) |
| 2013-2015 | 4109 (1.85) | 1525 (0.69) | 6.21 (6.02-6.40) | 2.29 (2.17-2.4) | 1.90 (1.81-1.99) |
| 2016-2019 | 7476 (3.40) | 2934 (1.33) | 5.63 (5.50-5.76) | 2.25 (2.17-2.33) | 2.13 (1.99-2.27) |
| **Any psychiatric condition^b^** |  |  |  |  |  |
| Yes | 12,851 (6.58) | 2795 (3.43) | 9.10 (8.94-9.26) | 6.11 (5.89-6.34) | 1.22 (1.17-1.28) |
| No | 27,624 (3.61) | 15,786 (1.80) | 4.75 (4.70-4.81) | 2.54 (2.50-2.58) | 1.63 (1.59-1.66) |
| **Chronic pain condition^c^** |  |  |  |  |  |
| Yes | 15,870 (3.70) | 5936 (1.79) | 5.33 (5.25-5.42) | 2.95 (2.88-3.03) | 1.45 (1.40-1.50) |
| No | 24,605 (4.63) | 12,645 (2.01) | 5.79 (5.72-5.86) | 2.72 (2.67-2.76) | 1.61 (1.57-1.65) |
| **Any co-medication^d^** |  |  |  |  |  |
| Yes | 22,674 (4.91) | 2367 (2.50) | 6.84 (6.75-6.93) | 4.38 (4.20-4.56) | 1.19 (1.13-1.24) |
| No | 17,801 (3.57) | 16,214 (1.87) | 4.56 (4.49-4.62) | 2.65 (2.61-2.69) | 1.61 (1.57-1.64) |
| **BZDR at initiation^e^** |  |  |  |  |  |
| BZD | 15,908 (4.40) | 6931 (1.92) | 5.93 (5.83-6.02) | 2.76 (2.7-2.83) | 1.59 (1.55-1.63) |
| Z-drugs | 24,567 (4.10) | 11,650 (1.95) | 5.41 (5.34-5.48) | 2.80 (2.75-2.85) | 1.55 (1.52-1.63) |

*Note*: For all covariates, p-values for interaction between subgroup HRs are *p*<0.001. For BZDR at initiation, p-value from Wald test is *p*=0.013.

^a^ All reported hazard ratios represent the results of fully-adjusted model for the demographically matched cohort

^b^ Includes psychiatric disorders (schizophrenia, schizotypal, and delusional disorders, and psychotic disorders, bipolar disorders, depressive disorders, anxiety disorders, obsessive-compulsive disorder, reaction to severe stress and adjustment disorders, dissociative, somatoform and other neurotic disorders, mental retardation, autism spectrum disorders, attention deficit/hyperactivity disorder, disruptive behaviour disorders), suicide attempt/self-injury, nonorganic sleep disorders and organic insomnia, if recorded in the National Patient Register between 1997 (the start of ICD-10) and the cohort entry date

^c^ Includes conditions related to chronic pain syndrome (back and neck pain, headache, arthritis, osteoarthritis, and joint pain, temporomandibular joint disorders, fibromyalgia, other musculoskeletal/connective tissue pain, gastro-esophageal reflux disease, irritable bowel syndrome, chronic fatigue syndrome, interstitial cystitis, and sexual pain), if recorded in the National Patient Register between 1997 (the start of ICD-10) and the cohort entry date

^d^ Includes dispensation records of antidepressants, centrally acting sympathomimetics, mood stabilisers, non-BZD-antiepileptics, antipsychotics, non-BZD-anxiolytics, non-BZDR-hypnotics/sedatives, non-opioid analgesics, and opioids, if dispensed within 3 months before the cohort entry, according to the Prescribed Drug Register

^e^ Type of medication (either any BZDs [N03AE, N05BA, or N05CD] or Z-drugs [N05CF]), which was collected at the first BZDR dispensation of in 2007-2019

*Abbreviation*: BZD, benzodiazepines; BZDR, benzodiazepines and benzodiazepine-related Z-drugs; ICD, International Classification of Diseases; IQR, interquartile range

**Supplementary Table S9**. **Cumulative BZDR dosage (in diazepam equivalent milligrams) during the first year after BZDR initiation and the risk of developing alcohol-related problems (in 900,605 BZDR-recipients versus 900,605 matched unexposed individuals) and drug-related problems (in 897,743 BZDR-recipients versus 897,743 matched unexposed individuals), with the follow-up starting from the second year after BZDR initiation**

| **Cumulative BZDR dosage during the first year**  **(in diazepam milligram equivalent, DME)^a^** | **No. of events, n (%)** | **Crude incidence rate (95% CIs), per 1000 person years** | **Fully-adjusted model,**  **HR (95% CI)** |
| --- | --- | --- | --- |
| **Alcohol-related problems** |  |  |  |
| 0 DME (ref – matched unexposed) | 14,551 (1.62) | 2.61 (2.56-2.65) | 1.00 |
| >0 - <300 DME | 1419 (6.44) | 8.48 (8.04-8.92) | 1.76 (1.56-1.99) |
| ≥300 - <900 DME | 4198 (2.56) | 3.89 (3.77-4.01) | 1.49 (1.42-1.57) |
| ≥900 - <2300 DME | 7852 (2.99) | 4.36 (4.27-4.46) | 1.61 (1.55-1.68) |
| ≥2300 DME | 17,453 (3.88) | 5.89 (5.8-5.98) | 2.19 (2.13-2.26) |
| **Drug-related problems** |  |  |  |
| 0 DME (ref – matched unexposed) | 6225 (0.69) | 2.96 (2.92-3.01) | 1.00 |
| >0 - <300 DME | 10,094 (1.86) | 5.16 (5.08-5.23) | 2.22 (2.13-2.31) |
| ≥300 - <900 DME | 5293 (2.63) | 6.60 (6.47-6.74) | 3.48 (3.26-3.71) |
| ≥900 - <2300 DME | 3746 (3.55) | 8.28 (8.07-8.5) | 5.64 (5.13-6.19) |
| ≥2300 DME | 2920 (5.80) | 10.73 (10.35-11.1) | 10.59 (9.22-12.17) |

*Note*: In this analysis, only individuals with the follow-up longer than one year since the cohort entry were included (i.e., those who during the first year did not develop the outcome of interest or were not censored due to death, emigration from Sweden, end of study or – if unexposed – change of the exposure status). Therefore, from the original 960,430 pairs in the demographically matched cohort, 59,825 (6.2%) and 62,687 (6.5%) matched pairs were excluded from the analyses of alcohol-related problems and drug-related problems, respectively.

^a^ To obtain the cumulative BZDR dosage in diazepam milligram equivalent for the first year after BZDR initiation for each BZDR-recipient, we collected information on all BZDR dispensations during that year and then summed up the defined daily doses for each collected prescription; next, we calculated diazepam milligram equivalent dose, using the scale (**Supplementary Table S4** details the scale) to standardize cumulative dosage given that BZRD with different relative potency were included. The obtained cumulative BZDR dosage in DME were then categorized in quartiles. Same as for other analyses, the reference group was represented by non-recipients.

*Abbreviation*: BZDR, benzodiazepines and benzodiazepine-related Z-drugs; CI, confidence intervals; DME, diazepam milligram equivalent; HR, hazard ratio**Supplementary Table S10**. **Associations of incident BZDR use with the risk of developing any drug-related problems stratified by study characteristics in 960,430 BZDR-recipients and 960,430 matched unexposed individuals in the demographically matched cohort**

|  | **No. of events, n (%)** | | **Crude incidence rate (95% CIs), per 1000 person years** | | **Fully-adjusted model^a^** |
| --- | --- | --- | --- | --- | --- |
|  | **BZDR-recipients** | **Matched unexposed** | **BZDR-recipients** | **Matched unexposed** | **HR (95% CI)** |
| **Sex** |  |  |  |  |  |
| Women | 14,809 (2.57) | 3520 (0.61) | 3.29 (3.24-3.34) | 0.88 (0.85-0.91) | 2.12 (2.04-2.21) |
| Men | 15,419 (4.02) | 4707 (1.23) | 5.55 (5.46-5.64) | 1.74 (1.69-1.79) | 2.12 (2.04-2.20) |
| **Age at cohort entry, years** |  |  |  |  |  |
| 10-29 | 14,717 (10.59) | 4270 (3.07) | 14.11 (13.88-14.34) | 4.22 (4.10-4.35) | 2.24 (2.15-2.33) |
| 30-64 | 13,110 (2.30) | 3166 (0.55) | 2.80 (2.75-2.85) | 0.74 (0.72-0.77) | 2.17 (2.08-2.26) |
| ≥65 | 2401 (0.96) | 791 (0.32) | 1.54 (1.48-1.60) | 0.55 (0.52-0.59) | 1.95 (1.79-2.13) |
| **Cohort entry year** |  |  |  |  |  |
| 2007-2009 | 11,920 (4.21) | 3534 (1.25) | 3.69 (3.62-3.75) | 1.26 (1.22-1.30) | 1.87 (1.80-1.96) |
| 2010-2012 | 8431 (3.58) | 2357 (1.00) | 4.10 (4.02-4.19) | 1.22 (1.17-1.27) | 2.03 (1.92-2.14) |
| 2013-2015 | 6134 (2.79) | 1518 (0.69) | 4.60 (4.49-4.72) | 1.16 (1.10-1.22) | 2.45 (2.29-2.61) |
| 2016-2019 | 3743 (1.69) | 818 (0.37) | 5.65 (5.47-5.83) | 1.23 (1.14-1.31) | 2.85 (2.61-3.10) |
| **Any psychiatric disorder^b^** |  |  |  |  |  |
| Yes | 12,766 (6.54) | 1743 (2.14) | 9.03 (8.88-9.19) | 3.78 (3.60-3.96) | 1.57 (1.49-1.66) |
| No | 17,462 (2.28) | 6484 (0.74) | 2.98 (2.93-3.02) | 1.04 (1.01-1.06) | 2.21 (2.14-2.29) |
| **Chronic pain condition^c^** |  |  |  |  |  |
| Yes | 12,406 (2.89) | 2807 (0.85) | 4.14 (4.07-4.21) | 1.39 (1.34-1.44) | 1.80 (1.72-1.88) |
| No | 17,822 (3.35) | 5420 (0.86) | 4.16 (4.10-4.22) | 1.16 (1.13-1.19) | 2.34 (2.25-2.42) |
| **Any co-medication^d^** |  |  |  |  |  |
| Yes | 20,136 (4.36) | 1341 (1.41) | 6.04 (5.96-6.12) | 2.46 (2.33-2.6) | 1.60 (1.51-1.69) |
| No | 10,092 (2.02) | 6886 (0.80) | 2.56 (2.51-2.61) | 1.12 (1.09-1.14) | 2.22 (2.15-2.29) |
| **BZDR at initiation^e^** |  |  |  |  |  |
| BZD | 11,797 (3.26) | 3154 (0.87) | 4.35 (4.27-4.43) | 1.25 (1.21-1.29) | 2.00 (1.93-2.06) |
| Z-drugs | 18,431 (3.08) | 5073 (0.85) | 4.03 (3.97-4.09) | 1.21 (1.18-1.25) | 2.17 (2.11-2.24) |

*Note*: For all covariates except sex, p-values for interaction between subgroup HRs are *p*<0.001. For BZDR at initiation, p-value from Wald test is *p*<0.001.

^a^ All reported hazard ratios represent the results of fully-adjusted model for the demographically matched cohort

^b^ Includes psychiatric disorders (schizophrenia, schizotypal, and delusional disorders, and psychotic disorders, bipolar disorders, depressive disorders, anxiety disorders, obsessive-compulsive disorder, reaction to severe stress and adjustment disorders, dissociative, somatoform and other neurotic disorders, mental retardation, autism spectrum disorders, attention deficit/hyperactivity disorder, disruptive behaviour disorders), suicide attempt/self-injury, nonorganic sleep disorders and organic insomnia, if recorded in the National Patient Register between 1997 (the start of ICD-10) and the cohort entry date

^c^ Includes conditions related to chronic pain syndrome (back and neck pain, headache, arthritis, osteoarthritis, and joint pain, temporomandibular joint disorders, fibromyalgia, other musculoskeletal/connective tissue pain, gastro-esophageal reflux disease, irritable bowel syndrome, chronic fatigue syndrome, interstitial cystitis, and sexual pain), if recorded in the National Patient Register between 1997 (the start of ICD-10) and the cohort entry date

^d^ Includes dispensation records of antidepressants, centrally acting sympathomimetics, mood stabilisers, non-BZD-antiepileptics, antipsychotics, non-BZD-anxiolytics, non-BZDR-hypnotics/sedatives, non-opioid analgesics, and opioids, if dispensed within 3 months before the cohort entry, according to the Prescribed Drug Register

^e^ Type of medication (either any BZDs [N03AE, N05BA, or N05CD] or Z-drugs [N05CF]), which was collected at the first BZDR dispensation in 2007-2019

*Abbreviation*: BZD, benzodiazepines; BZDR, benzodiazepines and benzodiazepine-related Z-drugs; ICD, International Classification of Diseases; IQR, interquartile range

**Supplementary Table S11**. **Sensitivity analysis within the demographically matched cohort using the new definition of drug-related outcomes (i.e., without dispensation records for opioid use disorders medication) in 960,430 BZDR-recipients and 960,430 matched unexposed individuals**

|  | **No. of events, n (%)** | | **Crude incidence rate (95% CIs), per 1000 person-years** | | **Minimally-adjusted model^a^** | **Adjusted**  **model^b^** | **Fully-adjusted model^c^** |
| --- | --- | --- | --- | --- | --- | --- | --- |
|  | **BZDR-recipients** | **Matched**  **unexposed** | **BZDR-recipients** | **Matched unexposed** | **HR (95% CI)** | **HR (95% CI)** | **HR (95% CI)** |
| Any drug-related problems | 26,816 (2.79) | 7126 (0.74) | 3.68 (3.64-3.72) | 1.06 (1.04-1.09) | 3.65 (3.56-3.75) | 2.25 (2.18-2.32) | 2.18 (2.11-2.24) |
| *By type of events^d^:* |  |  |  |  |  |  |  |
| Drug use disorders | 13,592 (1.42) | 2348 (0.24) | 1.86 (1.83-1.9) | 0.35 (0.34-0.36) | 5.55 (5.32-5.80) | 2.91 (2.77-3.06) | 2.79 (2.66-2.93) |
| Poisoning | 2388 (0.25) | 376 (0.04) | 0.33 (0.31-0.34) | 0.06 (0.05-0.06) | 6.09 (5.47-6.79) | 3.43 (3.05-3.86) | 3.38 (3.00-3.80) |
| Offences | 10,745 (1.12) | 4466 (0.47) | 1.47 (1.45-1.5) | 0.67 (0.65-0.69) | 2.38 (2.30-2.46) | 1.79 (1.72-1.87) | 1.75 (1.68-1.83) |
| Deaths | 737 (0.08) | 185 (0.02) | 0.10 (0.09-0.11) | 0.03 (0.02-0.03) | 3.72 (3.17-4.37) | 2.27 (1.89-2.71) | 2.17 (1.81-2.60) |

^a^ Conditioned on matching variables (birth year and month, sex, country of residence at the cohort entry)

^b^ Additionally adjusted for calendar year at the cohort entry, disposable family income, history of any psychiatric conditions, chronic pain conditions, concomitant dispensations of other psychotropic, antiepileptic, and analgesic medications, if dispensed within 3 months before the cohort entry, and history of maternal and paternal substance-related problems

^c^ Additionally adjusted for the events of any alcohol-related outcome if it was recorded during the study follow-up (i.e., controlling for drug-related problems in the analysis of alcohol-related problems and visa verse)

^d^ Some individuals who, by the new definition, were defined as having ‘any drug-related problems’ may have more than one type of the outcome event that occurred at the same day (multiple simultaneous outcomes appeared in 2% of BZDR-recipients and in less than 3% of references). As a result, those individuals may appear in more than one analysis of the specific type of outcome.

*Abbreviation*: BZDR, benzodiazepines and benzodiazepine-related Z-drugs; CI, confidence intervals; HR, hazard ratio

**Supplementary Table S12**. **Sensitivity analysis #2 with different adjustments and sensitivity analysis #3 where incident BZDR users are compared to the incident users of other (non-BZDR) anxiolytics, hypnotics, and sedatives.**

|  | **No. of events, n (%)** | | **Crude incidence rate**  **(95% CIs), per 1000 person-years** | | **Fully-adjusted model^a,c^** | **Fully-adjusted model^b,d^** |
| --- | --- | --- | --- | --- | --- | --- |
|  | **BZDR-recipients** | **Unexposed individuals** | **BZDR-recipients** | **Unexposed individuals** | **HR (95% CI)** | **HR (95% CI)** |
| **Demographically matched cohort** | | |  |  |  |  |
| Any alcohol-related problems | 40,475 (4.21) | 18,581 (1.93) | 5.60 (5.55-5.66) | 2.79 (2.75-2.83) | 1.56 (1.53-1.59)^a^ | 1.53 (1.50-1.57)^b^ |
| Any drug-related problems | 30,228 (3.15) | 8227 (0.86) | 4.15 (4.10-4.20) | 1.23 (1.20-1.25) | 2.11 (2.05-2.17)^a^ | 2.17 (2.10-2.25)^b^ |
| **Co-twin control cohort** | | |  |  |  |  |
| Any alcohol-related problems | 438 (3.64) | 170 (1.35) | 4.75 (4.31-5.20) | 1.89 (1.61-2.18) | 2.15 (1.76-2.61)^a^ | 2.30 (1.79-2.95)^b^ |
| Any drug-related problems | 360 (2.99) | 90 (0.72) | 3.88 (3.48-4.28) | 1.00 (0.79-1.20) | 2.78 (2.15-3.59)^a^ | 3.42 (2.37-4.93)^b^ |
| **Comparison with other (non-BZDR) anxiolytics, hypnotics, and sedatives** | | | | |  |  |
| Any alcohol-related problems | 26,543 (3.62) | 16,451 (2.74) | 6.34 (6.26-6.42) | 5.13 (5.05-5.21) | 1.56 (1.53-1.59)^c^ | 1.60 (1.57-1.63)^d^ |
| Any drug-related problems | 18,020 (2.46) | 12,850 (2.14) | 4.28 (4.21-4.34) | 3.99 (3.92-4.06) | 2.34 (2.28-2.39)^c^ | 2.23 (2.18-2.29)^d^ |

*Note*: For the sensitivity analysis #2, the demographically matched cohort with 960,430 BZDR-recipients and 960,430 matched unexposed individuals as well as the co-twin control cohort with 12,048 BZDR-recipients and 12,579 unexposed co-twins. For the sensitivity analysis #3, we compared 732,838 incident BZDR-recipients (singletons as in the demographically matched cohort and with no dispensation records of other (non-BZDR) anxiolytics, hypnotics or sedatives prior to the first BZDR dispensation in 2007-2019) to 599,526 incident recipients of other (non-BZDR) anxiolytics, hypnotics, and sedatives (singletons with the first dispensation record of other (non-BZDR) anxiolytics, hypnotics or sedatives in 2007-2019 and no BZDR dispensation record prior to that).

^a^ In the demographically matched cohort and the co-twin control cohort, this fully-adjusted modelling is the same as in the main analyses. This means that a history of psychiatric conditions and concomitant use of other medication are included in the model as two binary variables for ‘any psychiatric conditions’ and ‘any co-medications’. Same results are reported in **Table 1**. Here these results are included to ease the interpretation of the HRs (95% CI) obtained in the sensitivity analysis #2 where adjustment strategy was slightly modified (footnote ‘b’ below).

^b^ Sensitivity analysis #2: In the demographically matched and the co-twin control cohorts, the adjustment strategy was modified, i.e., instead of a combined binary variable for ‘any psychiatric conditions’, 13 separate variables, for one condition each, we included as ([1] Schizophrenia, schizotypal, and delusional disorders, and psychotic disorders, [2] Bipolar disorders, [3] Depressive disorders, [4] Anxiety disorders, [5] OCD, [6] Reaction to severe stress and adjustment disorders, [7] Dissociative, somatoform and other neurotic disorders, [8] Mental retardation, [9]ASD, [10] ADHD, [11] Disruptive behaviour disorders, [12] Suicide attempt /self-injury, and [13] Nonorganic sleep disorders and organic insomnia [as listed in **Supplementary Table S3**]). Also, a similar modification was made for a combined binary variable for ‘any co-medication’, instead of which 8 separate binary variables, one for each type of concomitant medication, were included in the model as ([1] Antidepressants, [2) Psychostimulants [centrally acting sympathomimetics], [3] Mood stabilisers, [4] Other antiepileptics [excluding benzodiazepine derivatives], [5] Antipsychotics, [6] Other anxiolytics [excluding benzodiazepine derivatives] and other hypnotics and sedatives [excluding benzodiazepine derivatives and Z-drugs], [7] Analgesics non-opioids, and [8] Opioids [as listed in **Supplementary Table S1**]).

^c^ Sensitivity analysis #3 (with adjustment strategy similar to the main analysis of the demographically matched cohort). This model is adjusted for birth year and month, sex, country of residence at the cohort entry, calendar year at the cohort entry, disposable family income, history of any psychiatric conditions, history of chronic pain conditions, concomitant dispensations of other medications within 3 months before the cohort entry [the only exclusion is concomitant dispensations of other (non-BZDR) anxiolytics, sedatives, and hypnotics], history of maternal and paternal substance-related problems, and the events of the ‘other’ outcome if it was recorded during the study follow-up (i.e., controlling for drug-related problems in the analysis of alcohol-related problems, if occurred during the follow-up, and vice versa).

d Sensitivity analysis #3 (with adjustment strategy same as in the sensitivity analysis #2). This model adjusted for all variables listed under footnote ‘c’, but with separate 13 covariates for a history of each psychiatric condition and with separate 7 covariates for each type of concomitant medication [the only exclusion is concomitant dispensations of other (non-BZDR) anxiolytics, sedatives, and hypnotics].

*Abbreviation*: ADHD, Attention Deficit/Hyperactivity Disorder; ASD, Autism spectrum disorders; BZDR, benzodiazepines and benzodiazepine-related Z-drugs; CI, confidence intervals; HR, hazard ratio; OCD, Obsessive-compulsive disorder;


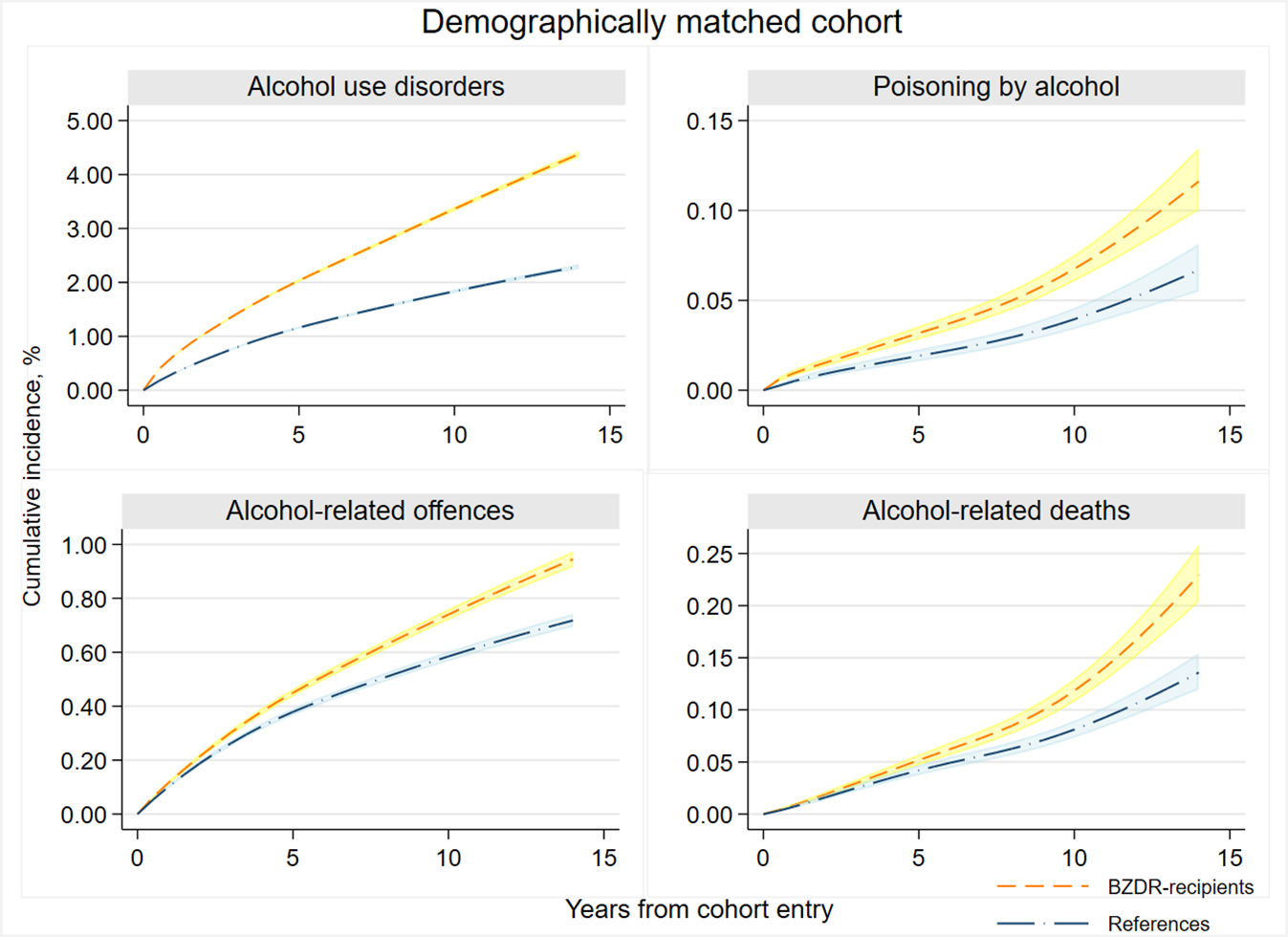


**Supplementary Figure S1**. **Standardized cumulative incidence and 95% CI of various types of incident outcome events within ‘any alcohol-related problems’ estimated as a function of time since the cohort entry among BZDR-recipients and their matched comparators (references) in the demographically matched cohort**

*Note*: Cumulative incidence measures are standardised, i.e., controlled for the covariates which were included in fully adjusted model for the demographically matched cohort, and estimated by the flexible parametric model. Shadowed areas denote 95% CI. The events indicated as ‘alcohol use disorders’ refer to the diagnoses and dispensations of medication for alcohol dependence.

*Abbreviations*: BZDR, benzodiazepine and benzodiazepine-related Z-drugs; CI, confidence intervals.


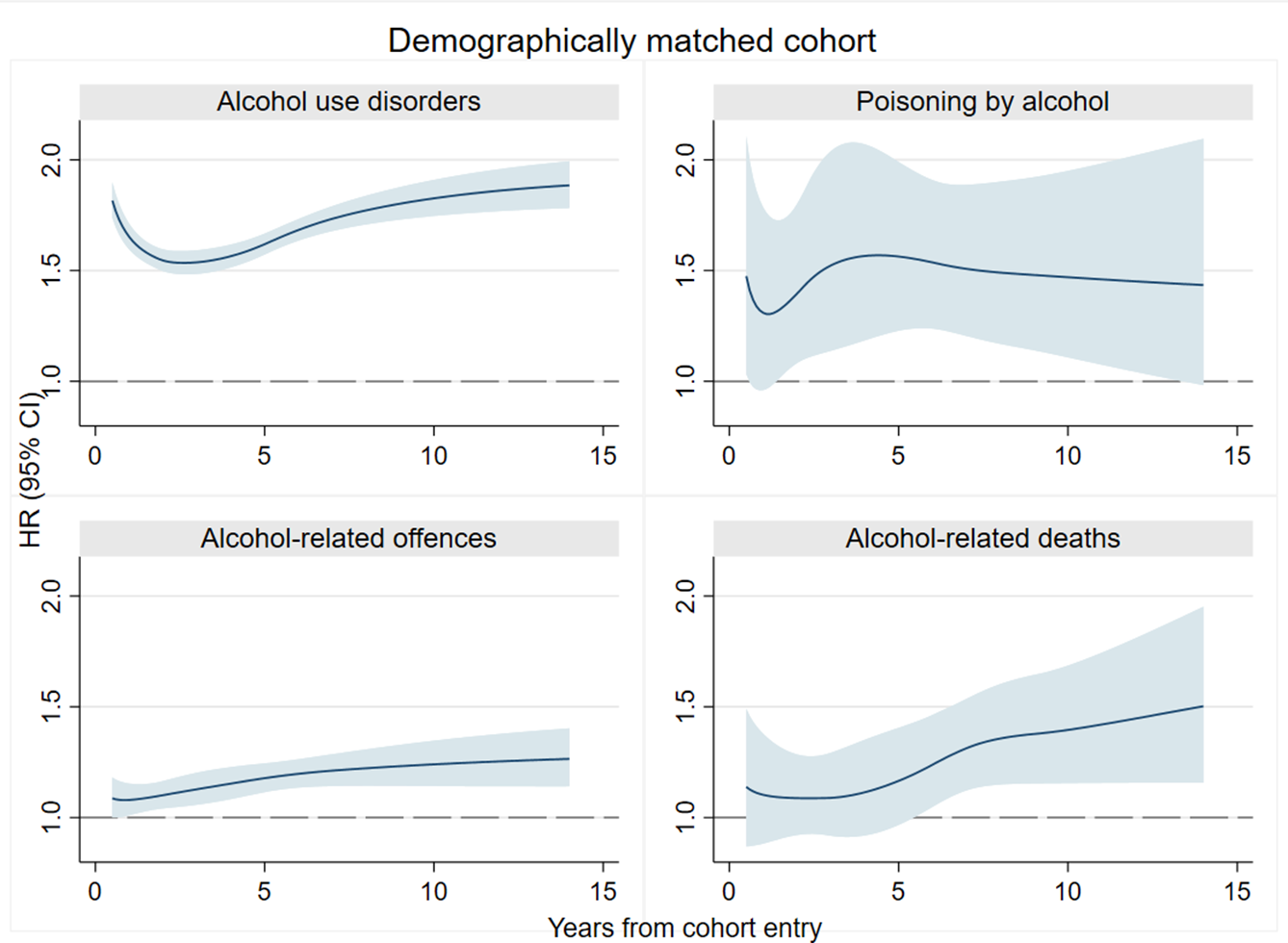


**Supplementary Figure S2**. **The risk of developing various types of incident outcome events within ‘any alcohol-related problems’ estimated as a function of time since the cohort entry in BZDR-recipients and their matched comparators (references) in the demographically matched cohort**

*Note*: All reported HRs represent the results from fully-adjusted model for the demographically matched cohort. Shadowed areas denote 95% CI. The events indicated as ‘alcohol use disorders’ refer to the diagnoses and dispensations of medication for alcohol dependence.

*Abbreviation*: BZDR, benzodiazepine and benzodiazepine-related Z-drugs; CI, confidence intervals; HR, hazard ratio


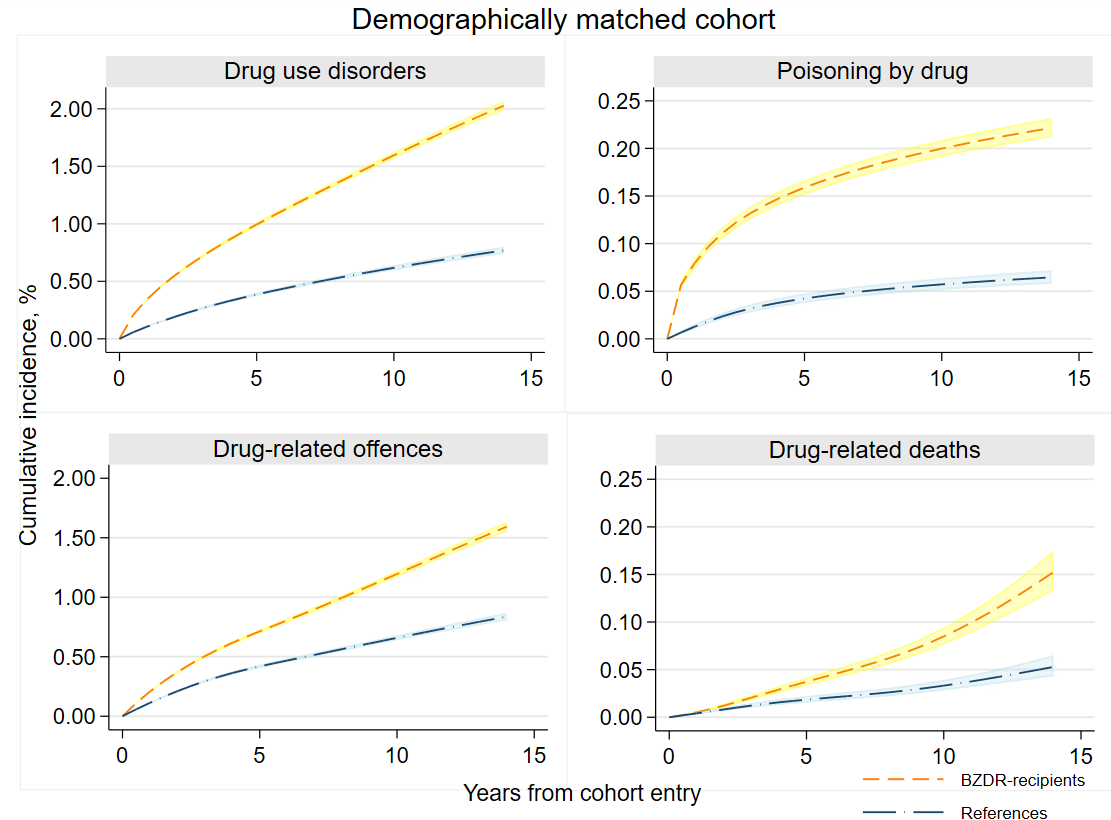


**Supplementary Figure S3**. **Standardized cumulative incidence and 95% CI of various types of incident outcome events within ‘any drug-related problems’ estimated as a function of time since the cohort entry among BZDR-recipients and their matched comparators (references) in the demographically matched cohort**

*Note*: Cumulative incidence measures are standardised, i.e., controlled for the covariates which were included in fully adjusted model for the demographically matched cohort, and estimated by the flexible parametric model. Shadowed areas denote 95% CI. The events indicated as ‘drug use disorders’ refer to the diagnoses and dispensations of medication for opioid use disorders.

*Abbreviations*: BZDR, benzodiazepine and benzodiazepine-related Z-drugs; CI, confidence intervals


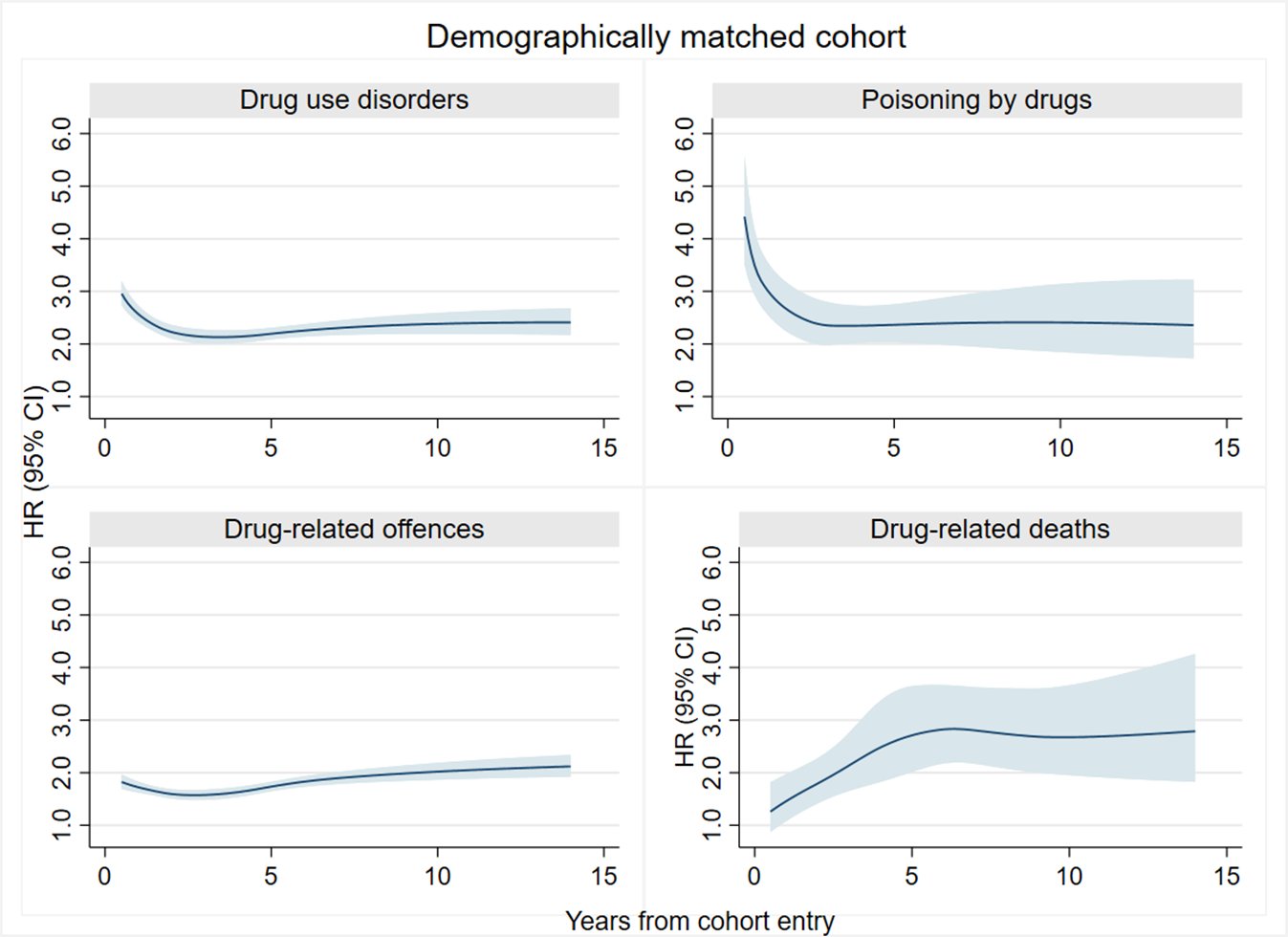


**Supplementary Figure S4**. **The risk of developing various types of incident outcome events as within ‘any drug-related problems’ estimated as a function of time since the cohort entry in BZDR-recipients and their matched comparators (references) in the demographically matched cohort**

*Note*: All reported HRs represent the results of fully-adjusted model for the demographically matched cohort. Shadowed areas denote 95% CI. The events indicated as ‘drug use disorders’ refer to the diagnoses and dispensations of medication for opioid use disorders.

*Abbreviation*: BZDR, benzodiazepine and benzodiazepine-related Z-drugs; CI, confidence intervals; HR, hazard ratio.
